# Chronic intake of 4-Methylimidazole induces Hyperinsulinemia and Hypoglycaemia *via* Pancreatic Beta Cell Hyperplasia and Glucose Dyshomeostasis

**DOI:** 10.1038/s41598-018-35071-6

**Published:** 2018-11-19

**Authors:** Balakrishnan Rekha, Ganesan Velmurugan, Allen J. Freddy, Sivakumar Anusha, Tharmarajan Ramprasath, Karuppusamy V. Karthik, Shanmugarajan Suresh, Prerna Kulshrestha, Gilles Mithieux, Alexander R. Lyon, Govindan Sadasivam Selvam, Subbiah Ramasamy

**Affiliations:** 1 Department of Molecular Biology, Cardiac Hypertrophy Laboratory, School of Biological Sciences, Madurai Kamaraj University, Madurai-625 021, Tamil Nadu, India; 2 DST Unit of Nanoscience & TUE, Department of Chemistry, Indian Institute of Technology, Chennai-600 036, Tamilnadu India; 3 Department of Zoology, Madras Christian College, Chennai-600 059, Tamilnadu India; 4 Center for Molecular and Translational Medicine, Georgia State University, Atlanta, GA 30303 USA; 5 Institut de la Santé et de la Recherche Médicale, U1213, Lyon, 69372 France; 6 Université de Lyon, Lyon, 69008 France; 7 Université Lyon 1, Villeurbanne, 69622 France; 8 NIHR Cardiovascular Biomedical Research Unit, Royal Brompton Hospital and Imperial College, London, United Kingdom; 9 Department of Biochemistry, School of Biological Sciences, Madurai Kamaraj University, Madurai, 625 021 Tamil Nadu India

## Abstract

Caramel colours are the preferential food colouring agent globally, reaches wide age groups through eatables. Colas, a sweetened carbonated drink are most common caramel coloured beverage and its consumption is linked with diabetes, obesity, pancreatic cancer and other endocrine disorders. A major by-product produced during caramelization is 4-methylimidazole (4-MEI) that is detected in noteworthy concentrations in colas and other beverages. Previous studies revealed the neurotoxic and carcinogenic potential of 4-MEI in animals at higher doses but the effect of 4-MEI at theoretical maximum daily intake dose on glucose homeostasis is unexplored. Here, mice treated with 4-MEI (32 µg/kg bodyweight/day) for seven weeks exhibited severe hypoglycaemia and hyperinsulinemia mediated by hyperplasia of pancreatic beta cells and induces metabolic alterations. On combinatorial treatment, 4-MEI suppressed the glucogenic potential of non-artificial sweeteners and promotes lipogenesis. Furthermore, increased levels of C-peptide, LDL-cholesterol and triglycerides were observed in the humans with regular intake of 4-MEI containing beverages. In summary, 4-MEI induced pancreatic beta cell hyperplasia and leads to disruption of glucose and lipid homeostasis. This study suggests the need for further assessment and reconsideration of the wide usage of 4-MEI containing caramels as food additives.

## Introduction

Nowadays, humans are attracted towards eatables by the sense of vision rather than by the sense of taste. Different natural and predominantly synthetic food colouring agents are being used and several of them are classified as potent toxicants possessing mutagenic and carcinogenic properties^1,2^. Caramel colours are one of the oldest and widely used synthetic food colouring agents and evolved as the most favourite food colour from children to old ages^3^. Colas are a class of caramel coloured, sweetened carbonated soft drinks and are the major dietary source of caramel colours in humans^3^. Several epidemiological and experimental studies showed the association of cola consumption with diseases like diabetes, obesity, cardiovascular diseases, cancer and other diseases^4^. Artificial sweeteners, phosphoric acid, caffeine and benzoate preservatives were considered as the agents for these negative impacts of colas^4,5^. 4-Methyl imidazole(4-MEI), an undesirable by-product produced during class III (ammonia caramel) and class IV (sulphite-ammonia caramel) caramelization process is detected at potentially toxic levels in colas^6^. It is also detected in other beverages (coffee, iced tea, blended whiskey, malt beverages, dark beers) and eatables (non-emulsified sauces, caramel-coloured syrups, dairy-based deserts, and ammoniated molasses) that are using class III and IV caramel colours^7,8^.

Foraging animals fed ammoniated feed exhibited convulsant activity including restlessness, bellowing, frothing at the mouth, and paralysis^9^. Similar neurological dysfunctions were noticed in mice and rat fed with 4-MEI containing diet^10,11^. 4-MEI induced neurotoxicity was mediated by impairment of electron transfer chain at complex II in mitochondria and elevated oxidative stress^12^. A two-year study at high dose of 4-MEI showed the development of alveolar/bronchiolar adenoma and carcinoma in both male and female mice, while increased incidence of leukaemia was observed only in female rats^10,11^. Based on these reports, International agency for research on cancer classified 4-MEI as a group IIB carcinogen (possibly carcinogenic to humans). No mutagenic activity of 4-MEI was observed on experiments with *Salmonella* and micronucleated erythrocytes^13^. All these carcinogenic and other studies were performed at doses of 4-MEI that far exceeded current estimates of human exposure to 4-MEI^10–13^. Hence, the present study is aimed to explore the effect of chronic intake of 4-MEI at dose equivalent to average daily intake by humans from dietary sources on animal physiology particularly glucose homeostasis and its consequences.

## Results

### Chronic intake of 4-MEI worsens glucose concentration in the circulation

**Figure 1:**
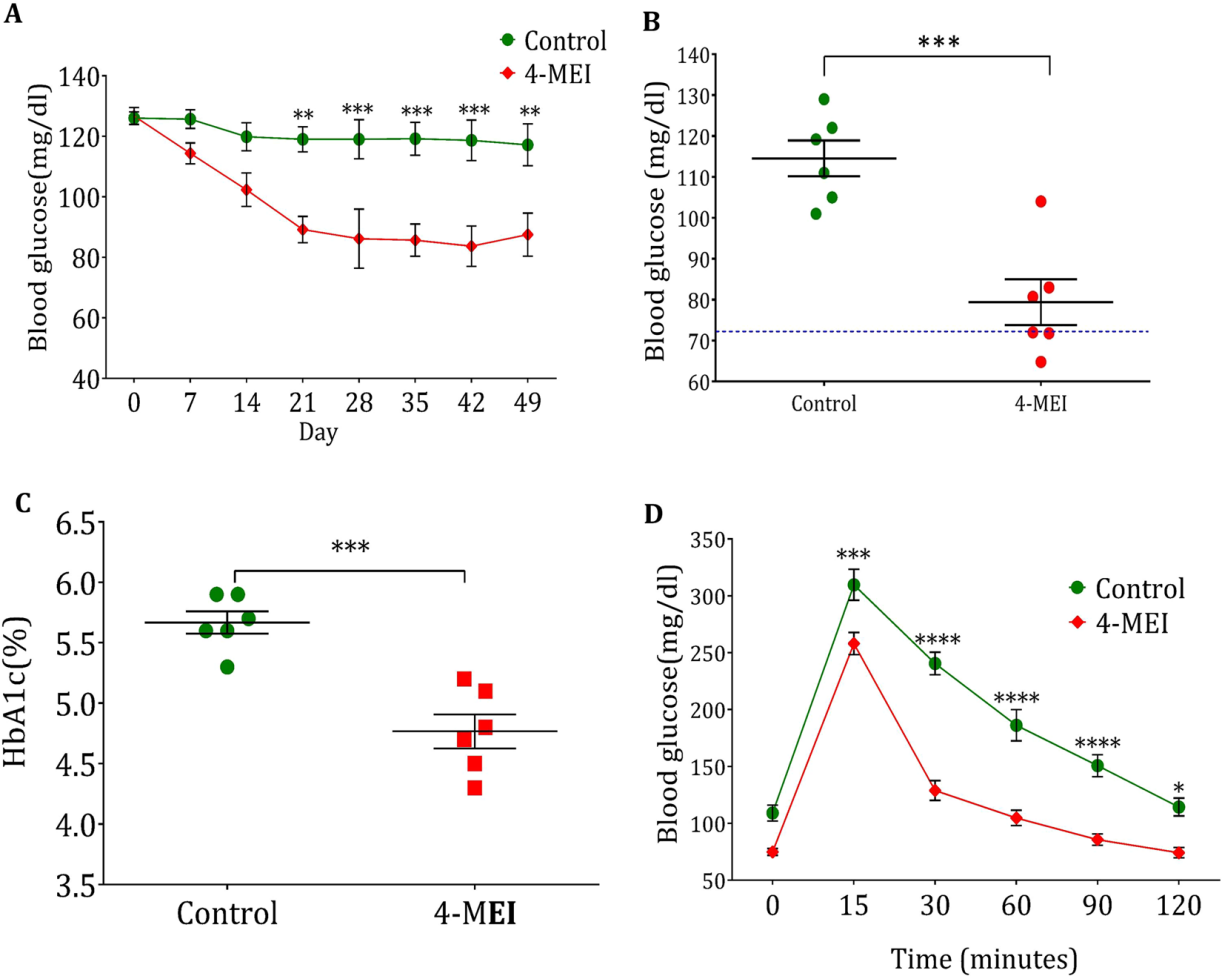
Chronic 4-MEI intake leads to hypoglycaemia in animals. (**A**) Periodical glucose level of control and 4-MEI treated animals. (**B**) Fasting blood glucose level on final day of treatment. (**C**) HbA1c level of control and 4-MEI treated animals. (**D**) Glucose clearance efficacy of experimental animals *via* OGTT. Error bars represent mean ± sem; ****P < 0.0001, ***P < 0.001, **P < 0.01, *P* < 0.05 for six animals in each group. Two-way ANOVA with Bonferroni correction and two-sided unpaired Student *t*-test. Experiments were repeated twice.

The maximum level of 4-MEI detected in beverages is 963.3 µg/l ^6^ and average consumption of 4-MEI containing beverages in Western countries is 200 ml per day. Theoretical maximum daily intake (TMDI) of 4-MEI by an average human being (60 kg body weight) is 3.2 µg/kg body weight. Since the exposure to 4-MEI through other beverages (coffee, iced tea, blended whiskey, malt beverages, dark beers) and eatables (non-emulsified sauces, caramel-coloured syrups, dairy-based deserts, and ammoniated molasses) are not considered for TMDI calculation, the mice were treated with 10X TMDI dose (32 µg/kg body weight) of 4-MEI orally using a gavage for seven weeks continuously. There was no significant change in body weight (*P* = 0.110; Supplementary Fig. S1A) and food intake (*P* = 0.054; Supplementary Fig. S1B). 4-MEI exposed mice exhibited a steady decrease in the level of blood glucose and reached hypoglycaemic condition after 35 days (*P* < 0.0001) and prolonged until the end of the experimental period (*P* < 0.001; Fig. 1A). Nearly 50% of the animals reached severe hypoglycaemia (level 1; Blood glucose < 70 mg/dl) at the end of the experiment (*P* < 0.001; Fig. 1B). A significant fall in the level of glycated haemoglobin (HbA1c) confirmed the hypoglycaemic condition (*P* < 0.001; Fig. 1C). Oral glucose tolerance test indicated high glucose clearance rate in the mice treated with 4-MEI (Fig. 1D; *P* < 0.0001 for AUC Supplementary Fig. S1C). The 4-MEI exposed mice reached normoglycemic condition (103 ± 3.8 mg/dl) in 30 minutes while control animals reached the normal glucose level (136 ± 5.3 mg/dl) only after 90 minutes (Fig. 1D). Collectively these experiments indicate that chronic exposure to 4-MEI at 10X TMDI dose induces hypoglycaemia with high metabolic glucose clearance rate.

### 4-MEI-induced hypoglycaemia is mediated by hyperinsulinemia

**Figure 2:**
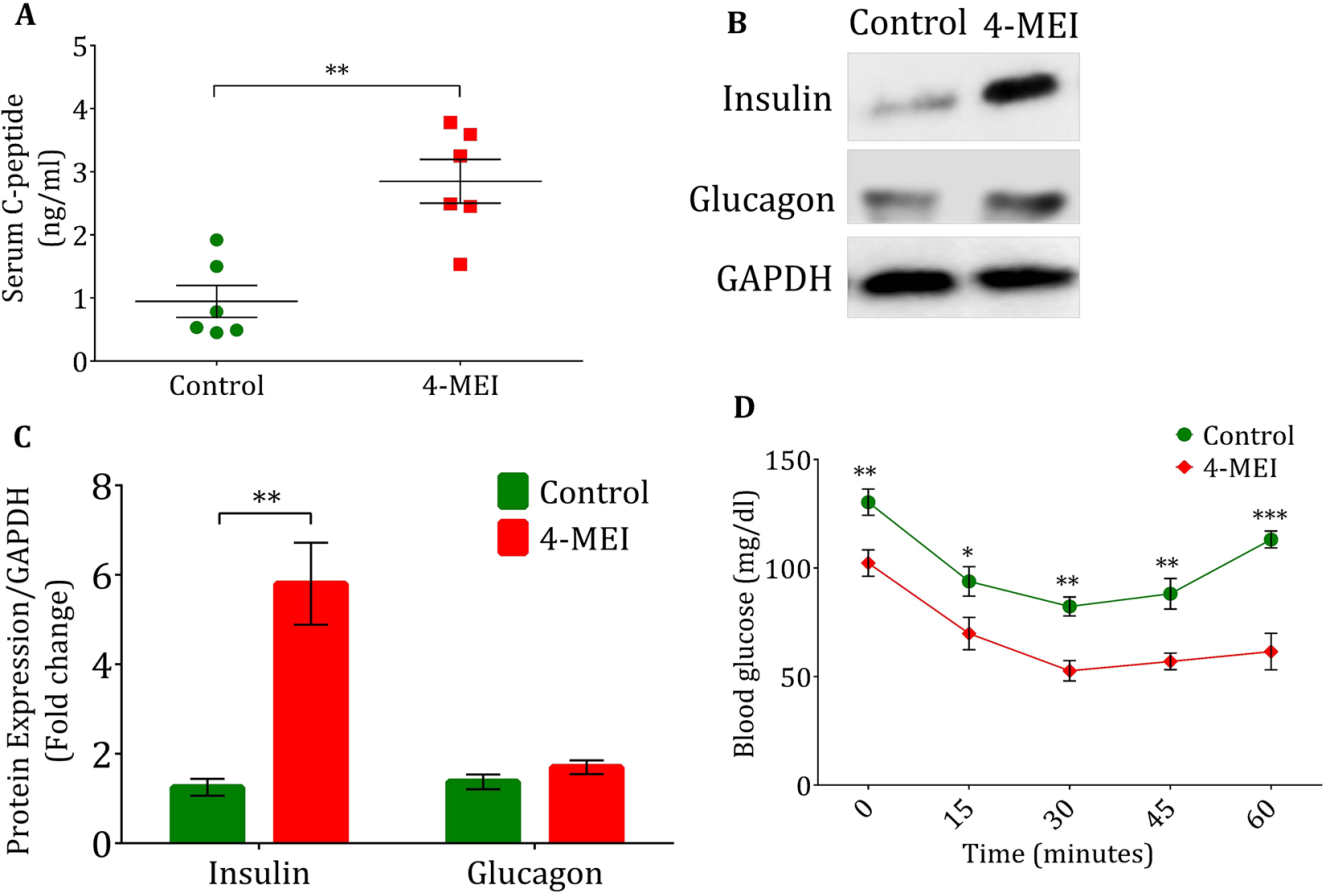
Hypoglycaemia mediated by 4-MEI is due to increased insulin secretion. (**A**) Serum C-Peptide levels of control and 4-MEI treated animals. (**B**) Immunoblot for glucagon and insulin in pancreatic tissue homogenate. (**C**) Quantitative data of glucagon and insulin immunoblots. (**D**) Determination of insulin sensitivity by ipITT. Error bars represent mean ± sem; ****P < 0.0001, ***P < 0.001, **P < 0.01, *P* < 0.05 for six animals in each group. Two-way ANOVA with Bonferroni correction and two-sided unpaired Student *t*-test. Experiments were repeated twice.

Insulin is the major hormone that lowers circulating glucose level and regulates glucose metabolism. Serum insulin C-peptide levels reflect endogenous insulin production and were increased in 4-MEI-fed animals indicating increased insulin synthesis (*P* < 0.01; Fig. 2A). In addition, glucagon and insulin expressions in the pancreatic tissue homogenates were quantified from immunoblotting studies (Fig. 2B,C). It showed significant increase in insulin expression (*P* < 0.0001), whereas there was no significant change in glucagon expression (*P* = 0.1163) of 4-MEI-fed animals compared to control. In 4-MEI exposed mice, insulin synthesis was nearly six times increased than glucagon (Fig. 2C). To evaluate insulin sensitivity and counteracting hormone function, intraperitoneal insulin tolerance test (ipITT) was performed. After insulin injection, blood glucose levels of 4-MEI-fed animals dropped significantly and did not recover from severe hypoglycaemia (level 2; blood glucose < 54 mg/dl) until 60 minutes. In contrast in control mice, glucose levels lowered after the insulin bolus, but the animals were never been hypoglycaemic (Fig. 2D). These results indicate that hypoglycaemia induced by 4-MEI was due to the increased insulin production by the pancreatic beta cells.

### Pancreatic beta cell hyperplasia induced by 4-MEI causes hyperinsulinemia

**Figure 3:**
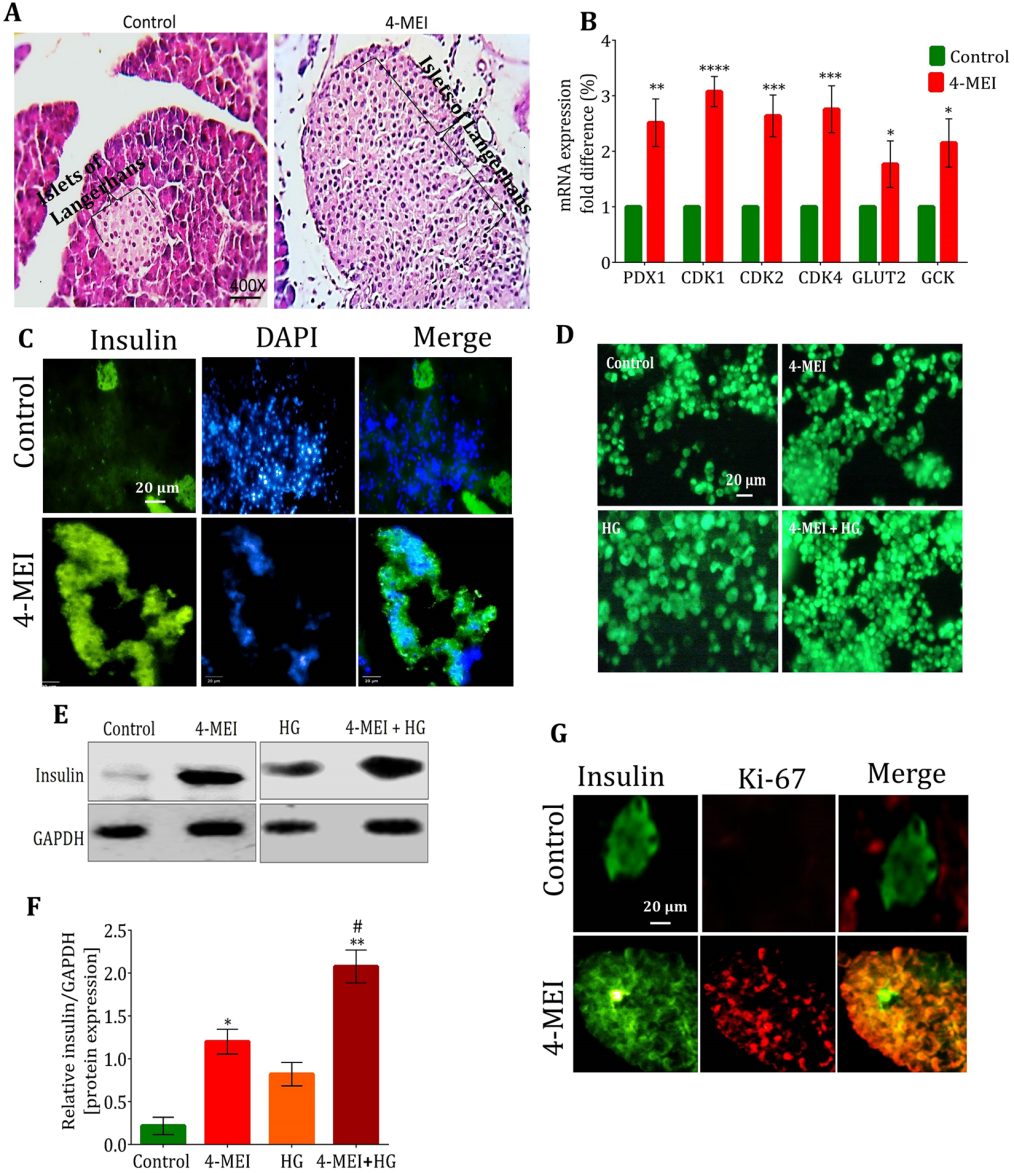
4-MEI induced increased insulin secretion is mediated by pancreatic islet cell hyperplasia. (**A**) Histopathology photomicrographs of pancreatic tissue sections of control and 4-MEI treated animals, stained with H & E (400×). (**B**) *In vivo* expression of islet cell specific and cell cycle regulatory genes by *q*PCR. (**C**) Determination of islet expansion and beta cell hyperplasia using anti-Insulin. (**D**) Acridine orange staining of MIN-6 cells. (**E**) *In vitro *protein expression of insulin. (**F**) Quantification of insulin expressions in *in vitro* systems. GAPDH served as housekeeping gene for normalization of mRNA/protein respectively. (**G**) Determination of beta cell proliferation and insulin secretion in pancreas tissue sections of experimental animals by IHC using anti-Ki-67 and aniti-Insulin. * is used to compare experimental groups with the control and # is used for the comparison between HG and 4-MEI+HG groups. Error bars represent mean ± sem; ****P < 0.0001, ***P < 0.001, **P < 0.01, *P* < 0.05/ ^####^*P* < 0.0001, ^###^*P* < 0.001, ^##^*P* < 0.01. Two-way and One-way ANOVA with Bonferroni correction. All *in vivo* experiments were repeated twice and *in vitro* experiments were performed with biological triplicates and technical duplicates.

Histopathological analysis of pancreas from 4-MEI exposed mice demonstrated an increase in the number of pancreatic endocrine cells (Figs 3A and S3A; *P* < 0.01). Augmented expression of beta cell functional [Pancreatic duodenal homeobox 1 (*PDX-1; P* < 0.01), glucose transporter (*GLUT2; P* < 0.05) and glucokinase (*GCK*; *P* < 0.05)] and proliferation markers such as Cyclin dependent kinases (*CDK1; P* < 0.0001, *CDK2; P* < 0.001*, CDK4; P* < 0.001) indicates hyperplasia of the beta cells (Fig. 3B). In 4-MEI-fed animals, all these genes studied showed more than two percent incremented expression. Subsequently, increased beta cell mass and insulin positive area in the pancreas of 4-MEI-fed animals revealed beta cell hyperplasia and increased insulin synthesis than control (Figs 3C and S3G). Further to validate the 4-MEI induced pancreatic beta cell hyperplasia, MIN-6 cells (pancreatic beta-cell line) were treated with 4-MEI (10 µg/ml for 48 hours). This concentration was selected based on cell viability test with different concentrations (Supplementary Fig. S3B). High glucose (33 mM D-glucose) was used as positive control. Staining with acridine orange (AO) (Fig. 3D) and crystal violet (CV) (Supplementary Fig. S3D) indicated significant cell proliferation with AO 4-MEI (*P* < 0.05), CV 4-MEI (*P* < 0.01) and AO 4-MEI+Glucose (*P* < 0.01), CV 4-MEI+Glucose *(P* < 0.01; Supplementary Fig. S3C,E) but not during high glucose treatment. The level of insulin is increased in both 4-MEI (*P* < 0.05) and 4-MEI+HG treatment (*P* < 0.001) (Fig. 3E,F). 4-MEI induced cell hyperplasia in MIN-6 cells was confirmed by the increased expression of genes associated with beta cell proliferation and function (Supplementary Fig. S3F). Significant upregulation of *PDX1*, *CDK1*, *CDK2, CDK4*, *GLUT2* and *GCK* genes were observed in 4-MEI and 4-MEI+HG treated cells. Whereas, HG treated cells showed incremented expression of *GLUT2* and *GCK* alone indicates no significant increase in cell number. But under high glucose milieu, to improve the glucose clearance rate, insulin, *GLUT2* and *GCK* expressions were moderately increased in HG treated cell than control. Subsequently, increased ki-67 positive cells and incremented insulin synthesis in the pancreatic islets of 4-MEI fed mice demonstrate pathological hyperplasia of the islet cells (Fig. 3G). In summary, 4-MEI induced hyperinsulinemia is not due to the increase in insulin synthesizing potential of existing cells but due to hyperplasia of beta cells.

### 4-MEI alters glucose and lipid metabolism

**Figure 4:**
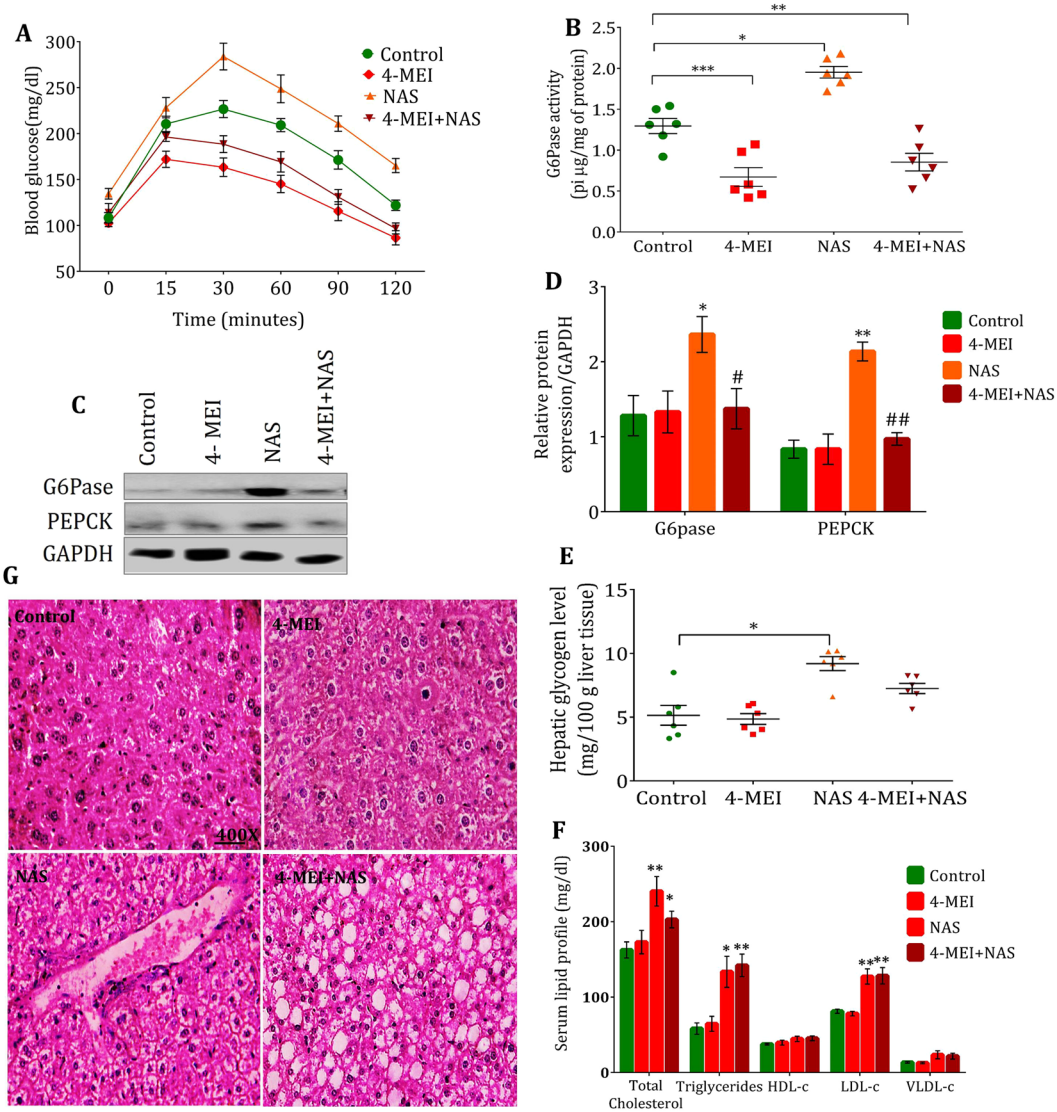
Combinatorial 4-MEI supresses glucose anabolism and promotes lipid synthesis. (**A**) Determination of gluconeogenesis rate during combinatorial treatment *via *ipPTT, (**B**) Activity of G6Pase in control and 4-MEI treated animals, (**C**) Representative immunoblot images of G6Pase and PEPCK in liver tissue during NAS, 4-MEI and combinatorial treatments, (**D**) Quantification of G6Pase and PEPCK expression. GAPDH served as the loading control for immunoblot. (**E**) Change in liver glycogen level during NAS, 4-MEI and combinatorial treatments, (**F**) Altered lipid profile during NAS, 4-MEI and NAS+4-MEI treatment, (**G**) Representative histopathology photomicrographs of hepatic tissue sections from NAS, 4-MEI and NAS+4-MEI treated animals stained with H & E (400×). * is used to compare experimental groups with the control and # is used for the comparison between NAS and 4-MEI+NAS groups. Error bars represent mean ± sem; ****P < 0.0001, ***P < 0.001, **P < 0.01, *P* < 0.05/ ^####^*P* < 0.0001, ^###^*P* < 0.001, ^##^*P* < 0.01. Two-way ANOVA with Bonferroni correction and two-sided unpaired Student *t*-test. Experiments were repeated twice.

In day-to-day life, humans are exposed to 4-MEI frequently in combination with non-caloric artificial sweeteners (NAS) through soft drinks. NAS are proved to induce glucose intolerance through augmented gluconeogenesis and lipogenesis and contribute to diabetes^14,15^. To mimic the natural conditions, the animals were co-treated with both 4-MEI and NAS (aspartame is used as prototypical NAS). Hyperglycaemia was observed in animals treated with only NAS (*P* < 0.01) whereas this was not seen in 4-MEI and 4-MEI+NAS treatment but instead they exhibited comparatively low sugar than control (*P* < 0.01; Supplementary Fig. S4A). Using intraperitoneal pyruvate tolerance test (ipPTT) circulatory glucose was not significantly raised by pyruvate in 4-MEI treated animals but, NAS treated animals showed elevated glucose level (*P* = 0.052), suggesting augmented glucose anabolism *via* gluconeogenesis (Figs 4A and S4B). In addition, glucose synthesis from gluconeogenesis was not significantly elevated in 4-MEI+NAS-treated animals indicating improved counteractions (*P* < 0.01; Figs 4B and S4B). Significantly reduced glucose-6-phosphatase (G6Pase; the rate limiting enzyme of gluconeogenesis) activity was observed in 4-MEI (*P* < 0.001) and combinatorial treatment (*P* < 0.01) while in NAS exposed mice, G6Pase activity was significantly increased than other groups (*P* < 0.05; Fig. 4B). On mRNA (Supplementary Fig. S4C) and protein expression analysis (Fig. 4C,D), G6Pase and other key enzymes [fructose-1, 6-bisphosphatase (FBPase), and phosphoenolpyruvate carboxykinase (PEPCK)] showed significant downregulation during 4-MEI and 4-MEI+NAS treatment and significant upregulation during NAS treatment compared to untreated control. The rate of gluconeogenesis was reflected on the liver glycogen content. Increased glycogen level was noticed during NAS treatment (*P* < 0.05) and the level was decreased during 4-MEI and 4-MEI+NAS treatment (Fig. 4E). In addition, 4-MEI+NAS treated animals showed significant elevated mRNA levels of GCK indicating improved glucose clearance (*P* < 0.01; Supplementary Fig. S4D). No significant effect of 4-MEI on lipid profile was noticed but 4-MEI+NAS combinatorial treatment induced elevated synthesis of total cholesterol, c-LDL and triglycerides (Fig. 4F). Subsequently, 4-MEI+NAS and NAS exposed mice demonstrate cytoplasmic fat droplets that displaying nucleus to the periphery in the hepatic tissue (Fig. 4G). Overall, during high glucose milieu 4-MEI inhibits glucose synthesis *via* gluconeogenesis and glycogenolysis but promote lipogenic potential of NAS through metabolic interconversions.

### 4-MEI does not induce oxidative stress and apoptosis

**Figure 5:**
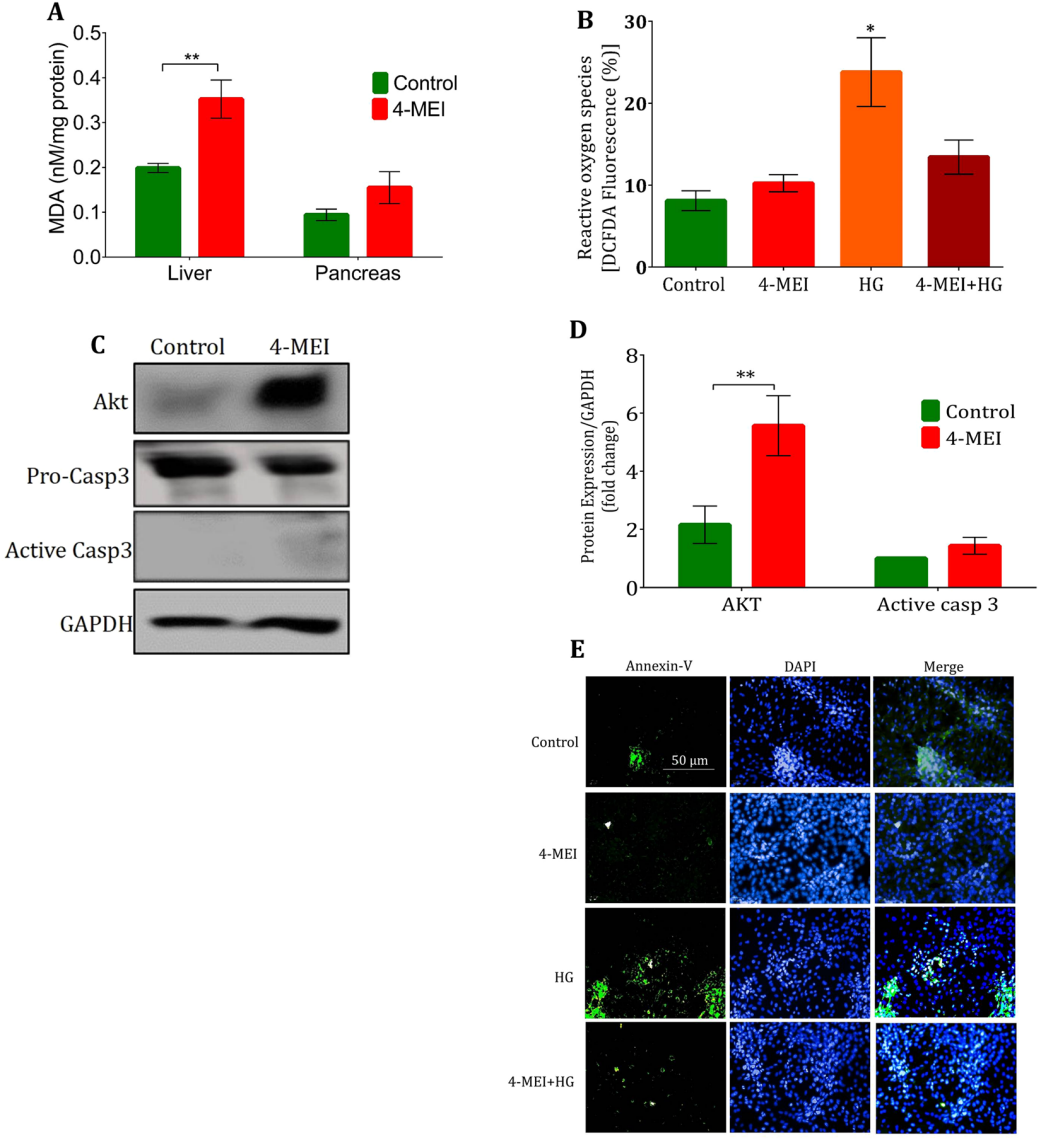
Chronic 10X TMDI dose of 4-MEI did not induce oxidative stress and cell death to islet cells. (**A**) Lipid peroxidation level in liver and pancreas, (**B**). *In vitro* ROS level, (**C**) Immunoblot for AKT and Caspase using pancreatic tissue lysate of experimental animals, (**D**) Quantitative data for AKT and Caspase immunoblots from pancreatic tissue lysate, (**E**) *In vitro* apoptosis determination by Annexin-V staining (200×). * is used to compare experimental groups with the control and # is used for the comparison between HG and 4-MEI+HG groups. Error bars represent mean ± sem; ****P < 0.0001, ***P < 0.001, **P < 0.01, *P* < 0.05/ ^####^*P* < 0.0001, ^###^*P* < 0.001, ^##^*P* < 0.01. One and Two-way ANOVA with Bonferroni correction. *In vivo* experiments were repeated twice or thrice and *in vitro* experiments were performed with biological triplicates and technical duplicates.

Disruption to glucose homeostasis produces reactive oxygen species (ROS) and induces oxidative stress leading to cell damage and apoptosis. Significant increases of lipid peroxidation were observed in liver tissue but not in pancreas during 4-MEI treatment (*P* < 0.01; Fig. 5A). On the other hand, no significant change in protein carbonyls level in both pancreas and liver (Supplementary Fig. S5A) was noticed. Increased level of total antioxidants was observed in both liver and pancreas (Supplementary Fig. S5B). In MIN-6 cells, 4-MEI exposure did not induce ROS (*P* = 0.25 Fig. 5B) and mitochondrial superoxide (mtO_2_^•−^) production (*P* = 0.08 Supplementary Fig. S5C,D) while, high glucose treatment leads to increased total ROS (*P* < 0.05; Fig. 5B) and O_2_^−^ levels (P < 0.001 Supplementary Fig. S5C,D) and this phenomenon was suppressed by 4-MEI on combinatorial treatment (Fig. 5B, Supplementary Fig. S5C,D).

The effects of oxidative stress on apoptosis and cell death were studied in both animals and cell line. 4-MEI significantly incremented the expression of Akt (*P* < 0.001) and no changes were observed in the expression of active Caspase 3 (*P* = 0.09; Fig. 5C,D). On histopath analysis, no signs of inflammation or cell death were noticed in pancreas (Fig. 3A) and liver tissues (Fig. 4G). In contrast NAS treatment induced periportal inflammation and pathological changes in hepatic tissue and on combinatorial treatment; 4-MEI suppressed these pathological changes (Fig. 4G). In MIN-6 cells, apoptosis was determined by annexin V (Figs 5E, S5E) and activated Caspase 3 expression (Supplementary Fig. S5F,G). 4-MEI not induced apoptosis but, increased annexin-V uptake and elevated expression of activated Caspase3 was observed during high glucose treatment and subsequent treatment of 4-MEI suppressed these effects (Fig. 5E, Supplementary Fig. S5E–G). Therefore, chronic 4-MEI exposure does not increase oxidative stress, apoptosis and cell death but induces pancreatic beta cell hyperplasia and disturbs glucose metabolism.

### Caramel coloured soft-drink consumption elevated insulin level in humans

**Figure 6:**
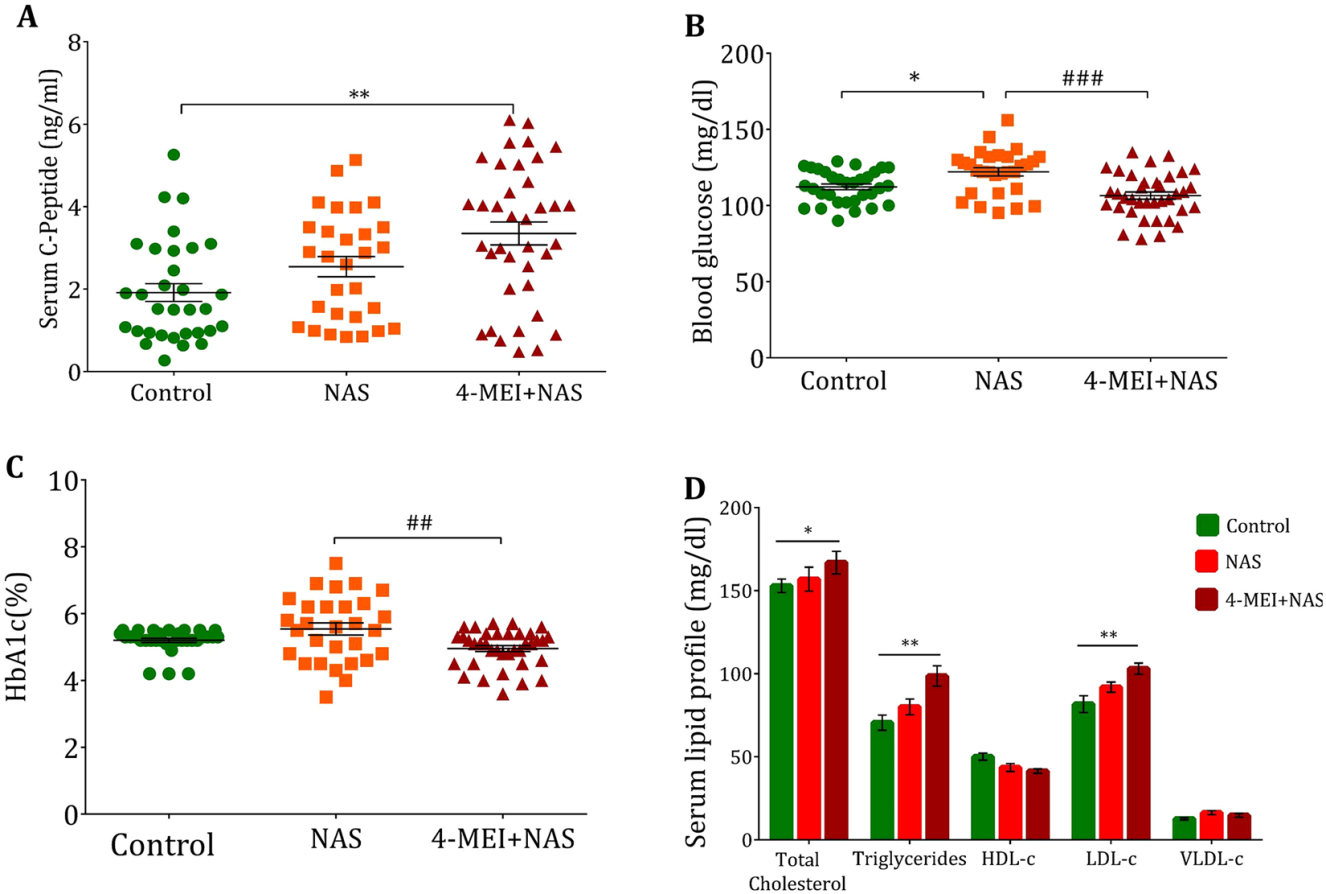
Chronic intake of 4-MEI through soft drinks induces hyperinsulinemia in humans. (**A**) Change in serum C-Peptide levels in NAS and 4-MEI+NAS consumers, (**B**) Fasting glucose level of NAS and 4-MEI+NAS consumers, (**C**) HbA1c level of NAS and 4-MEI+NAS consumers, (**D**) Lipid alteration induced during regular NAS and 4-MEI+NAS consumption. * is used to compare experimental groups with the control and # is used for the comparison between NAS and 4-MEI+NAS groups. Error bars represent mean ± sem; ****P < 0.0001, ***P < 0.001, **P < 0.01, *P* < 0.05/ ^####^*P* < 0.0001, ^###^*P* < 0.001, ^##^*P* < 0.01. One-way ANOVA with non-parametric Kruskal-Wallis test.

To validate the findings from animal and *in vitro* studies, blood samples were collected from human volunteers categorized into three groups based on their soft drink consumption behaviour. i) 4-MEI+NAS containing soft drinks consumers (4-MEI+NAS; n = 37), ii) NAS-only containing soft drinks consumers (NAS; n = 29) and iii) control (n = 32). Healthy volunteers consuming minimum of 04 servings per week (200 ml/day) a softdrink (NAS/4-MEI+NAS) are defined as soft drink consumers. The participants were within the age group of 18–25 years. Alcoholics, smokers and those with a familial history of hyperglycaemia/hypoglycaemia were excluded from the study. Ethical clearance was obtained from institute ethical committee and informed consent obtained from all participants. We observed a significant increase (*P* < 0.01) in the level of fasting C-peptide of 4-MEI+NAS cohort compared to control (Fig. 6A). Inspite of regular consumption of sugary drinks, we observed no significant difference in fasting blood glucose (*P* = 0.53) and HbA1c level (*P* = 0.24) of 4-MEI+NAS drink consumers (Fig. 6C,D) compared to control. Whereas, NAS consumers showed significant increase (*P* < 0.05) in fasting blood glucose (Fig. 6B) and moderate elevation in HbA1c level (Fig. 6C). In addition, we noticed a significant increase in total cholesterol (*P* < 0.05), LDL-cholesterol (*P* < 0.01) and triglyceride levels (*P* < 0.01) in serum of 4-MEI+NAS group (Fig. 6D). No significant changes were observed in the markers of liver and kidney damage (Supplementary Fig. S6A–C). Overall, these results suggest that 4-MEI induces insulin and cholesterol synthesis and overwhelms the glucogenic potential of NAS.

## Discussion

At present scenario, humans are becoming repositories to number of synthetic chemicals mainly *via* food products. Recently, researchers have raised concerns regarding 4-MEI toxicity in caramel coloured food products because of the frequent detection of 4-MEI levels in products with widespread distribution and increasing consumption. Carcinogenicity study in mice with 4-ME at daily dose of 40 to 170 mg/kg body weight revealed no difference in food intake but rats fed with 260 mg/kg body weight displayed decreased food consumption and both the group showed significant loss in the body weight^10,11^. 4-MEI containing class IV caramel consumption in mice for 13 weeks lowered food intake and significantly decreased body weight^16^. This is in concordance with the present study where there is a marginal decrease in the body weight of 4-MEI-fed animals without significant change in the food consumption at low chronic dose (Fig. S1). Though the dosage (10X TMDI) employed in this study is nearly 1000 fold less than previous studies^10,11^, but we still observed significant changes in the metabolism.

The last few decades have seen an increased mortality rate due to endocrine disorders and most of the synthetic chemicals including agrochemicals, food additives and heavy metals are reported as tremendous endocrine disturbing toxicants that particularly alters glucose homeostasis^17–21^. This promoted us to study the glycaemic changes induced during 4-MEI intoxication. In the present study, we observed that 4-MEI ingestion in mice suppresses circulatory glucose level by elevating glucose catabolism (Fig. 1A,B). 4-MEI fed animals were hypoglycaemic with increased glucose clearance efficacy which clearly shows that 4-MEI indeed promotes glucose catabolism (Fig. 1D). This is in contrast to most of the previously mentioned synthetic chemicals which were proven to induce hyperglycaemia and soft drink consumption is associated with diabetic prevalence^21–24^.

Since 4-MEI consumption in animals induces hypoglycaemia, the major hormones involved in glucose regulation were measured. 4-MEI fed animals showed about eight-fold elevated insulin expression with unaltered glucagon levels (Fig. 2). Further *in vivo* and *in vitro* study revealed the incidence of islet cell hyperplasia induced during 4-MEI exposure and insulin sensitivity was significantly elevated in 4-MEI fed animals than untreated controls. Earlier it was evidenced that pancreatic islet cell hyperplasia inappropriately increases insulin secretion and causes severe hypoglycaemia in humans^25^. Concurrently in *in vitro* study, 4-MEI treatment in a pancreatic beta cells increases glucose independent insulin secretion by promoting cellular hyperplasia whereas, in HG treated cells insulin secretion rises without increase in cell number.

NAS and 4-MEI are the major additives in the soft drinks. NAS are proven to induce hyperglycaemia in animals and humans by augmenting gluconeogenesis^26,27^ and based on our results, 4-MEI induces the opposite effect with hyperinsulinemic-hypoglycaemia in animals. In our human studies, the cohorts consuming 4-MEI also had suppressed glucose level compared to controls and NAS only consumers. In our preclinical *in vivo* model, during NAS treatment, we observed an incremented expression of G6Pase and PEPCK. This revealed increased glucose synthesis through gluconeogenesis and an elevated glycogen level in the liver tissue shows increased glycogenesis. In addition, elevated levels of cholesterol, triglycerides and LDL-c in serum of NAS treated animals clearly demonstrate the glucogenic and lipogenic potential of NAS. In contrast, 4-MEI potentially suppressed gluconeogenesis rate during ipPTT and also significantly lowered G6Pase activity, exerted a dominant effect over NAS and effectively controls glucose anabolism. In addition, 4-MEI+NAS treated animals also showed relatively decreased G6Pase activity and expression of G6Pase and PEPCK than NAS alone treated animals. Liver histological analysis displayed periportal inflammation with lipid droplets in NAS treated animals and found abundant lipid droplets that displacing nucleus to the periphery in 4-MEI+NAS treated animals. Together, these results demonstrate that, in the presence of elevated glucose milieu, 4-MEI mediates metabolic alterations and significantly promotes lipogenesis. Concomitantly, obesity, metabolic syndrome and during prediabetes insulin promotes lipogenesis, triglyceridemia and hepatic steatosis and further in the presence of high glucose milieu, insulin actively promotes liver glycogen synthesis and de novo lipogenesis^28,29^ was in accordance with our data.

Previous reports from Singapore, Chinese and American population strongly associated the link of pancreatic cancer with soft drink consumption and those studies focused upon the sugar load of the soft drinks studied^30,31^. An interesting observation in these studies was the higher prevelance of pancreatic cancer associated with cola soft drink consumers. This suggests that sugar is not alone the causative factor and our data raises the possibility that 4-MEI may be the culprit driving pancreatic beta cell hyperplasia even at the lower levels detectable in food and drinks consumed. 4-MEI is known to have a carcinogenic effect at much higher doses in preclinical studies^10^, and we now demonstrate that 4-MEI consumption at TMDI dose induces hyperplasia of the pancreatic islet cells, increases insulin synthesis and significantly suppresses glucose levels, with the potential to elevate lipogenesis in animals and in humans. Our study highlighted the probable pancreatic carcinogenic potential of 4-MEI but detailed investigations are needed to further confirm its toxicity, and specifically the progression of these proliferating cells into pancreatic tumors remains to be investigated. Today 4-MEI is widely used and a complete toxicological risk assessment including gut microbiota is the need of the hour^32^.

## Conclusion

This study demonstrates the hypoglycaemic and hyperinsulinemic effectsof 4-MEI mediated by the proliferation of pancreatic cells. Our results show the significant variation in glucose dyshomeostasis induced by 4-MEI, a toxic byproduct of caramel colour production which is frequently found in foods and drinks. These results show for the first time the potential toxic effects of 4-MEI at levels detectable in regularly consumed drinks and therefore clinically relevant. Our human studies support the findings from our *invivo* and *invitro* models. 4-MEI-induced pancreatic beta cell hyperplasia has concerning implications, and we believe it is important to revisit the epidemiological reports involving carbonated soft drinks consumers in general and recategorize the food and drink products as either 4-MEI-containing and non-4-MEI-containing, and reanalyze their association with diabetes, obesity, cancer and other diseases. Our results in combination with the other studies on 4-MEI toxicity, suggest the reassessment of the use of 4-MEI containing caramel colours and framing of regulations on 4-MEI based caramel colours usage in carbonated soft drinks and other foods.

## Methods

### Experimental animals

*Balb/c* mice were maintained in animal house at 24 ± 1 °C with 12-h day/night cycles and relative humidity of 45–60%. The animals were fed with deionized water and standard feed (Hindustan lever limited, India). All the experiments in this study were performed with female mice aged eight weeks weighing 20–28 g and maintained in a constant environment at 25–28 °C with 45–60% humidity. The health status of the mice was confirmed by continuously monitoring their activities, behaviour and body weight. The animal protocols used in this study were approved by the internal research and review board, ethical clearance, biosafety, and animal welfare committee of Madurai Kamaraj University (No. MKU-IRBEC-20/2014). The level of care provided to the animals met the basic requirements outlined in the guidelines formulated by National Institute of Health, USA.

### Experimental design and procedures

The mice were treated with 4-MEI (0.032 mg/kg body weight; Sigma-Aldrich Inc., USA; 822–36–6) or non-caloric artificial sweetener [7 mg/kg bodyweight; aspartame (Hi-Media Laboratories Pvt. Ltd, India; GRM1749)] or combination of both using oral gavage for seven weeks. The control animals were gavaged with equal volume of deionized water. Body weight, food and water intake of animals were monitored daily during the experiment. At the end of the experimental period, animals were anesthetized using ketamine (100 mg/kg body weight) and blood was harvested by cardiac puncture. Subsequently, organs including the brain, heart, liver, kidneys, large intestine and pancreas were dissected and perfused with sterile PBS and stored at −80 °C or in 10% formaldehyde.

### Fasting blood glucose & oral glucose tolerance test

Blood samples were collected by tail vein puncture from overnight fasted animals and the blood glucose was measured using biosensor-based glucometer (One Touch, Select^TM^ Life Scan, Europe 6300 Zug Switzerland). Glucose clearance rate was determined by oral glucose tolerance test (OGTT), performed by administering 15% glucose solution to overnight fasted animals (1.5 g/kg body weight). Tail vein blood samples were collected at 0, 15, 30, 60, 90 and 120 minutes after glucose administration and glucose levels were estimated using glucometer. Areas under the curves (AUC) for OGTT were calculated to evaluate glucose tolerance.

### Intraperitoneal insulin tolerance test

Insulin sensitivity and counter regulatory responses were determined by insulin tolerance test (ipITT)^33^. After 4 hours of fasting, animals were given intraperitoneal insulin injection (0.75U/kg body weight). Blood samples were collected at 0, 15, 30, 45 and 60 minutes through tail-vein puncture and glucose was estimated using glucometer. AUC for ITT were calculated to evaluate insulin sensitivity.

### Intraperitoneal Pyruvate Tolerance Test

Rate of gluconeogenesis was determined by pyruvate tolerance test (ipPTT). Briefly, sodium pyruvate (1.5 g/kg body weight) (Sigma-Aldrich Inc., USA; P2256) was injected *via* intraperitoneal injection to 16 hours fasted animals. Tail vein samples were collected at 0, 15, 30, 60, 90 and 120 minutes after pyruvate injection and glucose level was measured using glucometer. The areas under the curve of glycaemia vs. time were calculated to estimate the total glucose synthesized from pyruvate.

### Blood investigations

HbA1c level in whole blood was determined by HPLC (D10, Biorad Inc., USA). Serum and plasma samples were prepared from whole blood appropriately by standard protocols. The level of insulin was determined by measuring C-peptide level in the serum using commercial kit (Bio Vision Inc., USA; K4757-100) as per recommended protocols. Whole lipid status in serum was measured using commercial kits (Diasys Diagnostics Systems Gmbh, Germany & Agappe Diagnostics, India) as per manufacturer’s protocol. Expression of lactate dehydrogenase (LDH) (Teco diagnostics, Inc., USA), Aspartate transaminase (AST) and Alanine transaminase (ALT) (Siemens, Inc., USA) were quantitated in serum as per the recommended protocols by manufacturers.

### Histopathology and immunohistochemistry

Pancreatic and liver tissues were fixed with 10% formaldehyde and paraffin embedded by standard methods. Subsequently, the tissues were sectioned (5 µm thin) and stained with Hematoxylin & Eosin (H & E) and were mounted by mounting medium. At least 5 islets in each section were randomly chosen and analyzed. The cell number of each islet was quantified with Image J software (https://imagej.nih.gov/ij/). Tissue morphology was examined and analysed by clinical pathologist.

Pancreatic tissue sections were subjected to immunohistochemistry for insulin and glucagon using anti-Insulin (Santa Cruz Biotechnology USA; sc- 25840), anti-glucagon (Santa Cruz biotechnology USA; sc-74825) and anti-Ki-67 (Santa Cruz biotechnology USA; sc-101861) antibodies and HRP conjugated secondary antibody (Santa cruz Biotechnology USA; sc-2030) as per standard protocols. The sections were counter stained with Harris Hematoxylin (Nice Co., India) and the detection was based on the intensity of brown chromogen produced by HRP-DAB at the site of enzyme activity (3, 3′-diaminobenzidine) and the images were captured using light microscope. Subsequently for fluroscence imaging, anti-rabbit IgG-FITC (Sigma-Aldrich Inc., USA; F6005) and anti-mouse IgG-TRITC (Sigma-Aldrich Inc., USA; T5393) were used.

### Preparation of tissue homogenate and protein estimation

100 mg of the tissue was homogenized in ice cold RIPA buffer (Sigma-Aldrich Inc., USA; R0278) supplemented with protease inhibitors (Sigma-Aldrich Inc., USA; P8340) The tissue homogenate was spun at 12,000 rpm for 15 min at 4 °C and the supernatant collected was stored at −80 °C.The concentration of protein was estimated by Bradford assay^34^ (Sigma-Aldrich Inc., USA; B6926) using the BSA as a standard with technical triplicates.

### Glucose 6-phosphatase Assay

Concentration of inorganic phosphorus (Pi) released was quantified by Taussky-Shorr method^35^. Briefly, 100 µl of 200 mM Glucose 6-Phosphate (Sigma-Aldrich Inc., USA; G7879) was mixed with 150 µl of 100 mM tris buffer (pH: 6.5) and incubated at 37 °C for 5 minutes. Subsequently, tissue homogenate (10 µl) was added and incubated at 37 °C for 5 minutes. Ten percent of TCA was added and incubated at 25 °C for 5 minutes to terminate the reaction. Finally, the mixture was centrifuged at 5000 rpm for 10 min and supernatant was collected. Supernatant or inorganic Pi solution (Sigma Aldrich Inc., USA; P3869) was mixed with equal volume of Taussky-Shorrcolour reagent and incubated at 25 °C for 5 min. Finally, the absorbance was read at 660 nm and glucose 6-phosphatase (G6Pase) activity was expressed as µg of pi released per mg of protein. The experiments were performed with technical triplicates.

### Glycogen assay

Liver tissue (100 mg) was homogenized in 500 µl of TCA (3%) and the homogenate was centrifuged at 3000 rpm for 5 minutes. The supernatant was mixed with 5 volumes of ice cold ethanol (95%) and kept overnight at 20 °C for the precipitation of glycogen. After a spun, the supernatant was discarded and pellet was dissolved in 250 µl of deionized water. Blank and glucose standards (0.5 mg/ml) were prepared with same volume of deionized water. About 1.25 ml of Anthrone reagent (50 mg anthrone, 1 g thiourea, 72 ml H_2_SO_4_ in 100 ml deionized water) was added to all tubes and incubated at 95 °C for 15 minutes followed by cooling. Absorbance was measured at 620 nm and the amount of glycogen (mg/100 g of tissue) = (DU/DS) × 0.1 × (Volume of extract/gram of tissue) × 100 × 0.9. Where DU = absorbance of samples and DS = absorbance of glucose standard^36^. The experiments were performed with technical triplicates.

### Lipid peroxidation assay

The lipid peroxidation was measured by estimating malondialdehyde (MDA) levels^37^. Briefly, 100 μL of tissue homogenate was mixed with 200 μL of ice cold 10% TCA and incubated in ice for 15 min to precipitate the protein. The samples were centrifuged at 2500 rpm for 15 min at 4 °C and supernatant was mixed with equal volume of 0.67% thiobarbituric acid (TBA) and incubated in boiling water bath for 10 min. 1,1,3,3′-tetramethoxypropane (TMP) was used as the standard. The absorbance was read at 532 nm and the amount of MDA was expressed as nM/mg protein. The experiments were performed with technical triplicates.

### Protein carbonylation assay

Dinitrophenylhydrazine (DNPH) method^38^ was used to measure the carbonyls produced by oxidation of proteins. Briefly, serum/tissue homogenates (100 μL) were mixed with 400 μL of 10 mM DNPH dissolved in 2.5 M HCl and incubated for 1 h and the protein was precipitated with an equal volume of 10% TCA. The pellet obtained was washed with 1:1 ethanol:ethyl acetate mixture and suspended in 250 μL of 6 M guanidine HCl. The protein hydrozones were measured by reading OD at 370 nm. The concentration of protein carbonyls (nM) was determined as follows: [(CA)/0.011] [250/100]. CA is the corrected absorbance, calculated by measuring the difference between each sample and corresponding control. The reactions were performed with technical triplicates.

### Total antioxidant assay

Total antioxidant assay in the serum/tissue was performedusing the total antioxidant kit (Sigma-Aldrich Inc., USA; CS0790) as per the manufacturer’s protocol. The reactions were performed with technical triplicates and antioxidant concentration in the sample was expressed in mM qualified to the concentration of the Trolox standard.

### Cell culture

MIN6 cells were obtained from National Centre for Cell Science (NCCS), Pune, India. Cells were routinely maintained in complete media (Dulbecco’s modified Eagle’s medium (DMEM F-12) supplemented with 10% fetal bovine serum (FBS), 100 U/ml penicillin and 0.1 mg/ml streptomycin). Chemicals used for *in vitro* study were purchased from Sigma-Aldrich (St Louis, MO, USA). Cell culture media and consumables were obtained from Hi-Media Laboratories Pvt. Ltd (Mumbai, India). Cell passages ranging from 20–35 were used for this study and the cells were cultured in 95% air and 5% CO2 at 37 °C.

### Cell viability assessment by MTT assay

Cell viability was evaluated by measuring 3-(4, 5-dimethylthiazol-2-yl)-2, 5-diphenyltetrazolium bromide (MTT; Sigma-Aldrich Inc., USA; M2003) reduction. Cells were plated in 96-well plates (3 × 10^3^ per well) and allowed to attach overnight. Then, they were treated with medium containing various concentrations of 4-MEI (5, 10, 20, 50,100 μg) for 24, 48 and 72 h. After incubation, culture media was changed to serum-free media containing MTT (0.5 mg/mL). Media was removed after 4 h and DMSO was added to each well. The absorbance was measured at 570 nm.

### *In vitro* treatment

Cells were categorized in to four groups: 4-MEI treated (cells maintained in complete media were given 10 μg 4-MEI/ml for 48 h), high glucose (HG) treated (cells maintained in complete media were given 33 mM D-glucose for 12 h) to mimic glucotoxicity, 4-MEI+HG treated (cells maintained in complete media were given 33 mM D-glucose for 12 h and then treated with 10 μg 4-MEI for 48 h) and cells without any treatment served as the untreated control.

### Assessment of Cell number

After treatment, wells were washed with PBS, followed by the addition of acridine orange [0.1 mg/ml in ethanol]. The stained cells were examined under fluorescent microscope and cell number was determined based on the fluorescent intensities.

In addition, cell hyperplasia was predicted separately using crystal violet. Briefly, after PBS wash, the cells were fixed with 100% ice cold methanol followed by the addition of crystal violet (0.5% in methanol). After 10 minutes of incubation, cells were washed, dried and viewed under microscope. Atleast four fields from the each experimental condition was randomly selectedand the images were quantified using Image J software (https://imagej.nih.gov/ij/).

### Cellular Staining and imaging

After appropriate treatment, cells were washed twice with 1X PBS and stained with appropriate dyes. The cells were stained with 2′,7′ –dichlorofluorescin diacetate (DCFDA; Santa Cruz biotechnology, USA; sc-359840) and Mitosoxred (Thermo fisher scientific, USA; M36008) to determine reactive oxygen species and mitochondrial superoxide production respectively as per standard protocols. Apoptosis was determined by staining with Alexa fluor 488 Annexin V/Dead cell apoptosis kit (Molecular probes Inc., USA) and 4′, 6-Diamidino-2-Phenylindole, Dihydrochloride (DAPI; Thermo fisher scientific, USA.D1306) as per manufacturer’s guidelines. After appropriate staining and washing with 1X PBS, the cells were observed in fluorescence microscopy. Minimum of four fields from the each experimental condition was randomly selected and fluorescent intensity was quantified using Image J software

### Measurement of intracellular ATP content

ATP content in the cells were measured using the ATP Bioluminescence Assay Kit (Biovision, USA. Catalog No. K254), as per manufacturer’s protocol.

### Gene expression analysis

RNA from mice pancreas, liver or MIN6 cells was extracted with the single step TRI Reagent (Sigma–Aldrich Inc. USA) and cDNA was constructed using MMLV-reverse transcriptase (Thermoscientific™, USA). The *q*RT-PCR was performed with SYBR Green PCR master mix (MBI-Fermentas, Lithuania). DNA oligos used were synthesized by Eurofins Genomics India Ltd. (Bangalore, India). The oligos used in this study are listed in Supplementary Table 2. Real time cycling parameters include: Initial denaturation (95 °C-5 min), cycling [denaturation (95 °C-20 s), annealing (58 °C-20 s), and finally extension (68 °C-20 s), No of cycles – 40], followed by a melting curve analysis. The Ct values were corrected by Ct readings of corresponding internal control (*GAPDH*). Data from three determinations (mean ± SEM) were expressed as relative expression level. The reactions were performed in Master cycler Ep Realplex2 System (Eppendorf AG, Germany). The specificity of the reaction was confirmed by agarose gel electrophoresis.

### Protein Expression Analysis

Proteins extracted from cells and animal tissues were separated by SDS–Polyacrylamide gel electrophoresis (SDS-PAGE) and electroblotted on to PVDF membrane (Millipore, IPVH00010) following standard protocols. Primary antibodies used in this study were anti-Insulin (Santa Cruz Biotechnology USA; sc- 25840), anti-Glucagon (Santa Cruz biotechnology USA; sc-74825), anti-G6PASE-α (Santa Cruz Biotechnology USA; sc- 25840), anti-PEPCK-C (Santa Cruz biotechnology USA; sc-74825), anti-AKT (Santa Cruz Biotechnology USA; sc-8312), anti-CASPASE 3 (Pierce USA; PA5–16335) and anti-GAPDH (Santa Cruz Biotechnology USA; sc-25778) and secondary antibody used was HRP conjugated anti-rabbit IgG (Santa cruz Biotechnology USA; sc-2030). Chemiluminescence detection was performed with the chemiluminescence detection kit (Amersham^TM^ Western Blotting Detection Reagents, UK) according to the manufacturer’s instructions. The protein bands in western blots were quantified using Image J software.

### Human blood collection and biochemical analysis

5 ml of blood samples were collected in EDTA-coated and non-EDTA coated tubes from healthy volunteers (n = 98) in age group of 18 to 25. Smokers, alcoholics and those with familial history of hyperglycaemia/hypoglycaemia were eliminated from the study. The details of age, sex, height, weight, occupation, dietary habits, frequency of intake of 4-MEI containing and other beverages including tea and coffee and disease history and medications were collected using a standard questionnaire. Volunteers with the habit of regular intake (minimum 04drinks per week) were classified as soft drink consumers. They were categorized as 4-MEI+NAS (n = 37; regular intake of soft drinks containing both 4-MEI and NAS), NAS (n = 29; regular intake of non-caramel soft drinks containing NAS but not 4-MEI) and control (n = 32; no habit of intake of soft drinks). The sample collection protocol was approved by ethical clearance, biosafety and animal welfare committee of Madurai Kamaraj University (No. MKU-IRBEC-08/2015). Informed consent was obtained from all participants included in the study. All experimental procedures comply with the Helsinki Declaration. The level of serum glucose, HbA1c, total lipid profile, AST, ALT and serum creatinine were determined using the commercially available kits described previously.

### Statistics

The following statistical analyses were used for animal studies: a two-way ANOVA with Bonferroni post-hoc analysis was used to compare between groups in different time-points and one-way ANOVA with Tukey’s posthoc analysis or unpaired two-sided Student t-test was used to compare either between multiple or between two groups, respectively. In all relevant panels, symbols, bars, or horizontal lines represent the mean and error bars represent sem. For mouse experiments, cohort sizes match common practice of the described experiments and are repeated twice or thrice. For human experiments, sample size was chosen to validate statistical analyses. For human studies, the non-parametric Kruskal-Wallis test was employed. No data points were excluded from analyses in mice or human studies. P < 0.05 was considered statistically significant in all analyses.

## Electronic supplementary material

Supplementary figures
