# Supplementary material for: Chronic intake of 4-Methylimidazole induces Hyperinsulinemia and Hypoglycaemia via Pancreatic Beta Cell Hyperplasia and Glucose Dyshomeostasis
Source: Sci Rep. 2018 Nov 19;8:17037. doi: 10.1038/s41598-018-35071-6 (PMC6242838; doi:10.1038/s41598-018-35071-6)
Supplement: Supplementary file 1 — Supplementary figures [file 41598_2018_35071_MOESM1_ESM.pdf]

Supporting Information

Chronic intake of 4-Methylimidazole induces Hyperinsulinemia and Hypoglycemia *via* Pancreatic Beta Cell Hyperplasia and Glucose Dyshomeostasis

Rekha Balakrishnan[1], Velmurugan Ganesan[2], Allen J. Freddy[3], Anusha Sivakumar[1], Tharmarajan Ramprasath[4], Karuppusamy V. Karthik[1], Suresh Shanmugarajan[1], Prerna Kulshrestha[1], Gilles Mithieux[5], Alexander R. Lyon[6], Govindan Sadasivam Selvam[7] Subbiah Ramasamy[1]

[1]Cardiac Hypertrophy Laboratory, Department of Molecular Biology, School of Biological Sciences, Madurai Kamaraj University, Madurai 625 021, Tamilnadu, India.

[2]DST Unit of Nanoscience & TUE, Department of Chemistry, Indian Institute of Technology Madras, Chennai 600 036, Tamilnadu, India.

[3]Department of Zoology, Madras Christian College, Chennai, Tamilnadu, India.

[4]Center for Molecular and Translational Medicine, Georgia State University, Atlanta, GA 30303, USA.

[5]Institut de la Santé et de la RechercheMédicale, U855, Lyon 69372, France; Université de Lyon, Lyon 69008, France; Université Lyon 1, Villeurbanne 69622, France.

[6]NIHR Cardiovascular Biomedical Research Unit, Royal Brompton Hospital and Imperial College, London, United Kingdom.

[7]Department of Biochemistry, School of Biological Sciences, Madurai Kamaraj University, Madurai 625 021, TamilNadu, India.

Correspondence and requests addressed to S.R (subbiahr@nrcbsmku.org)

S1A

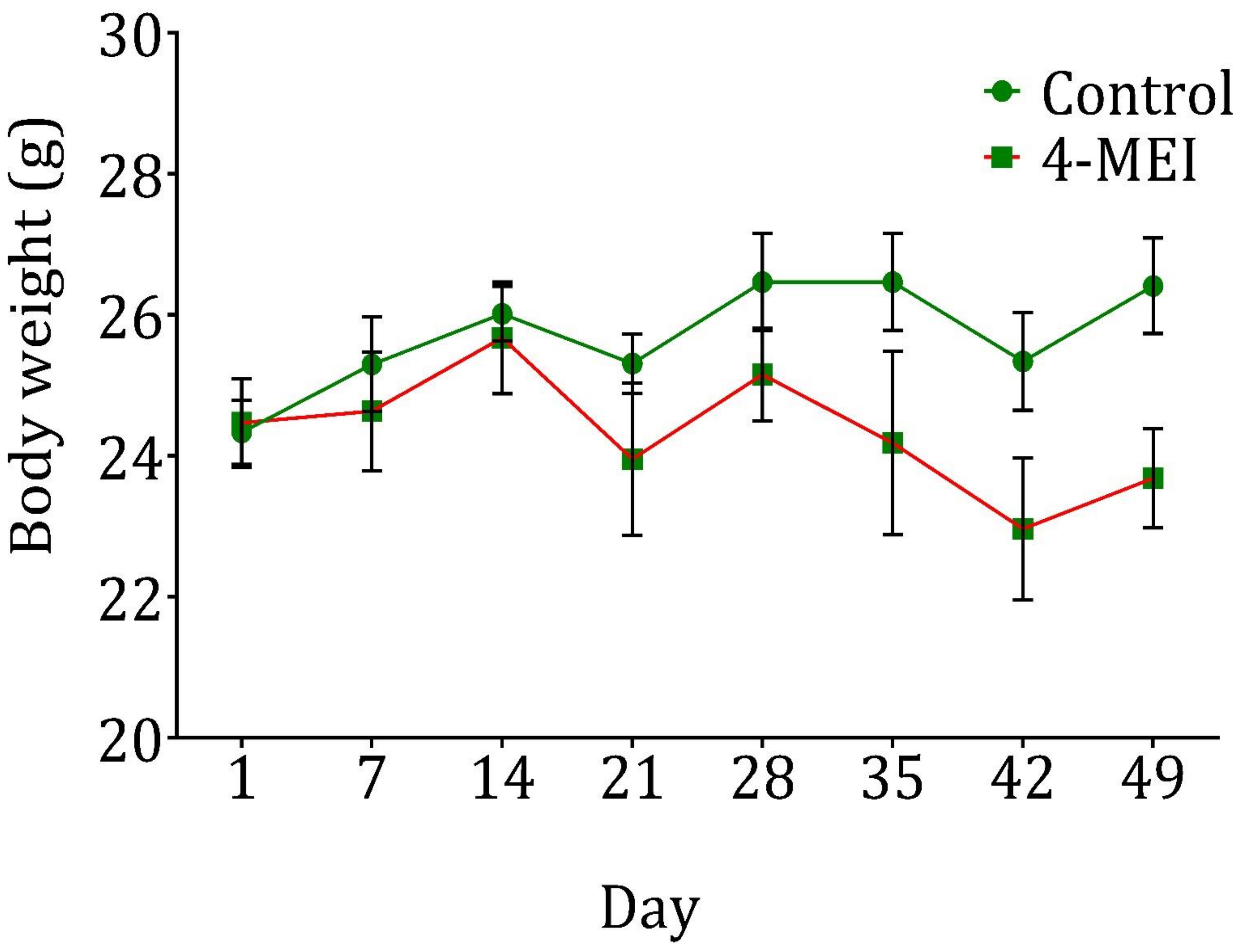

S1B

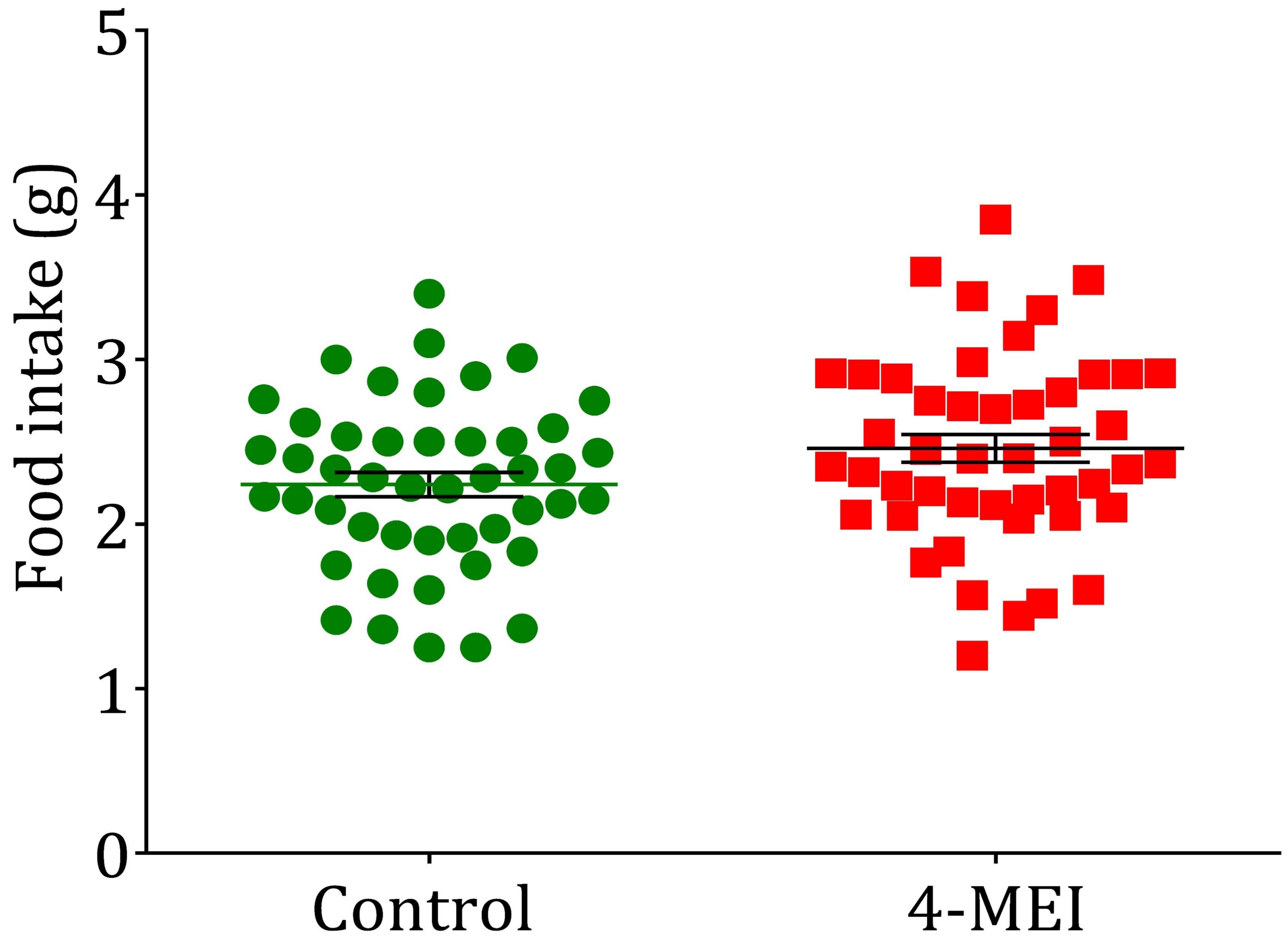

S1C

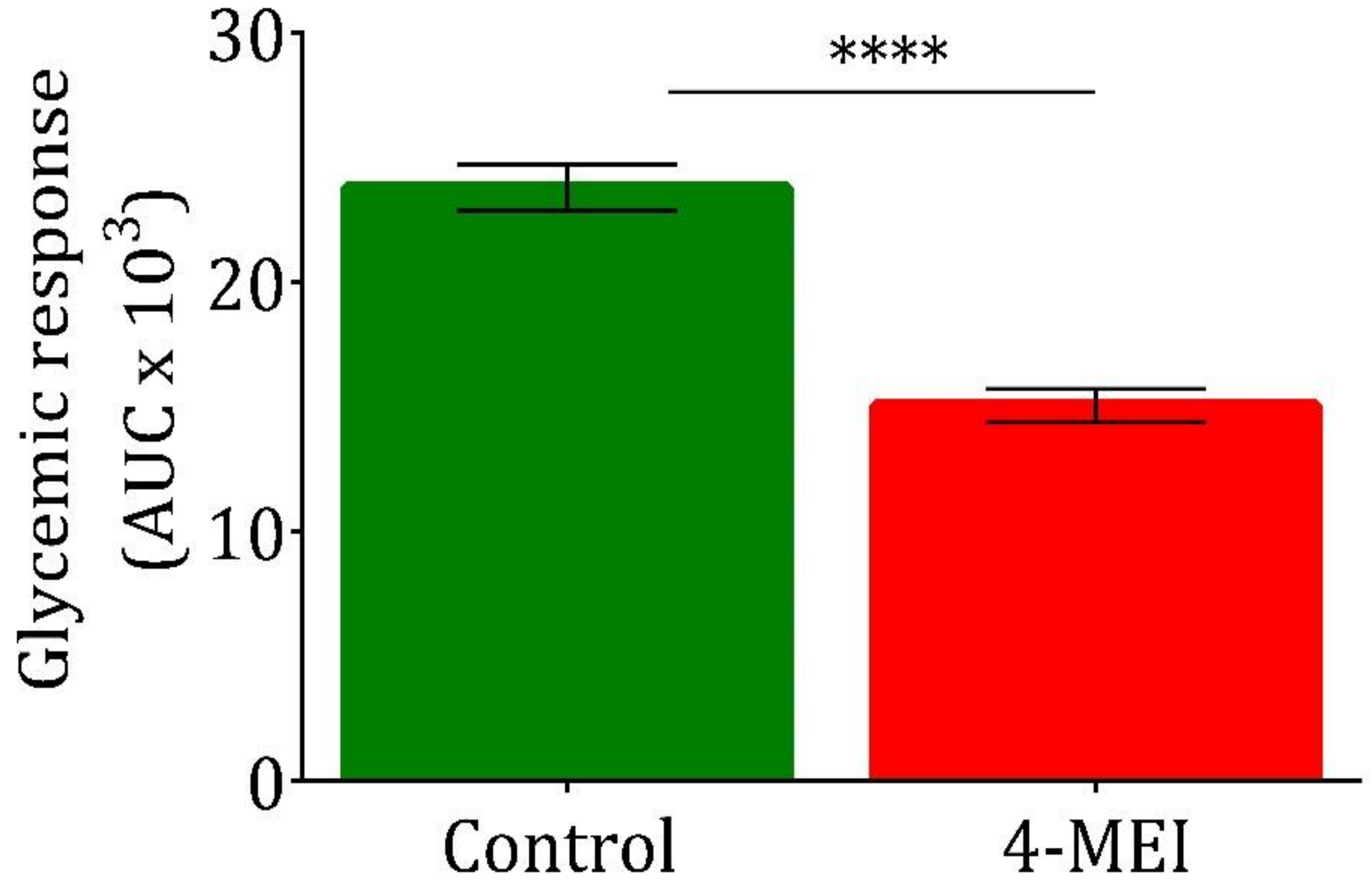

Supplementary Fig. 1: 4-MEI induced physiological changes and increased glucose catabolism. S1A. Body weight alterations of the mice during 4-MEI treatment, S1B. Change in food intake during chronic 4-MEI ingestion, S1C. In vitro AUC for OGTT. Error bars represent mean ± sem; **** P< 0.0001, *** P<0.001, **P< 0.01, P< 0.05. Two-way ANOVA with Bonferroni correction and two-sided unpaired Student *t*-test.

S2A

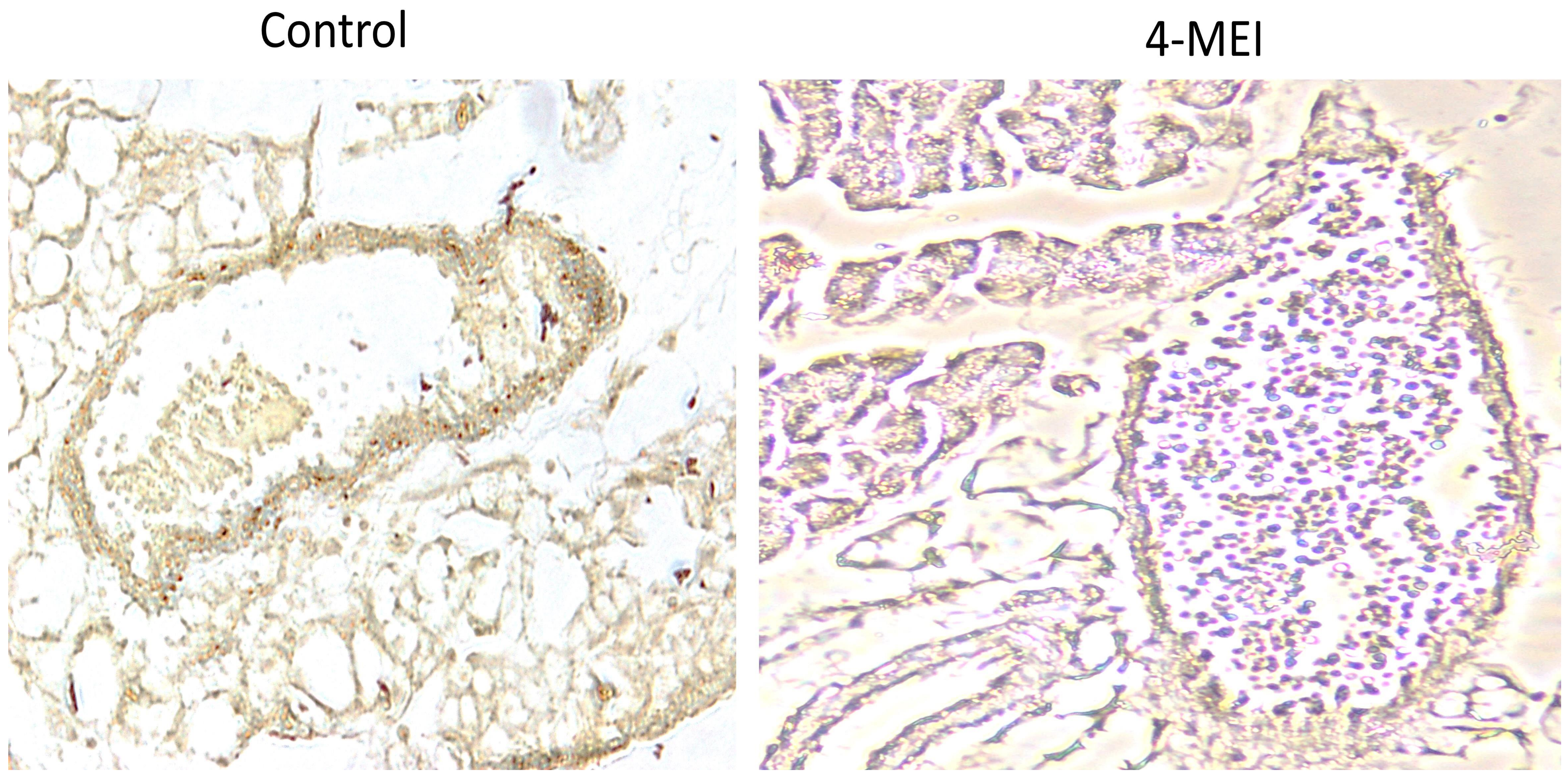

S2B

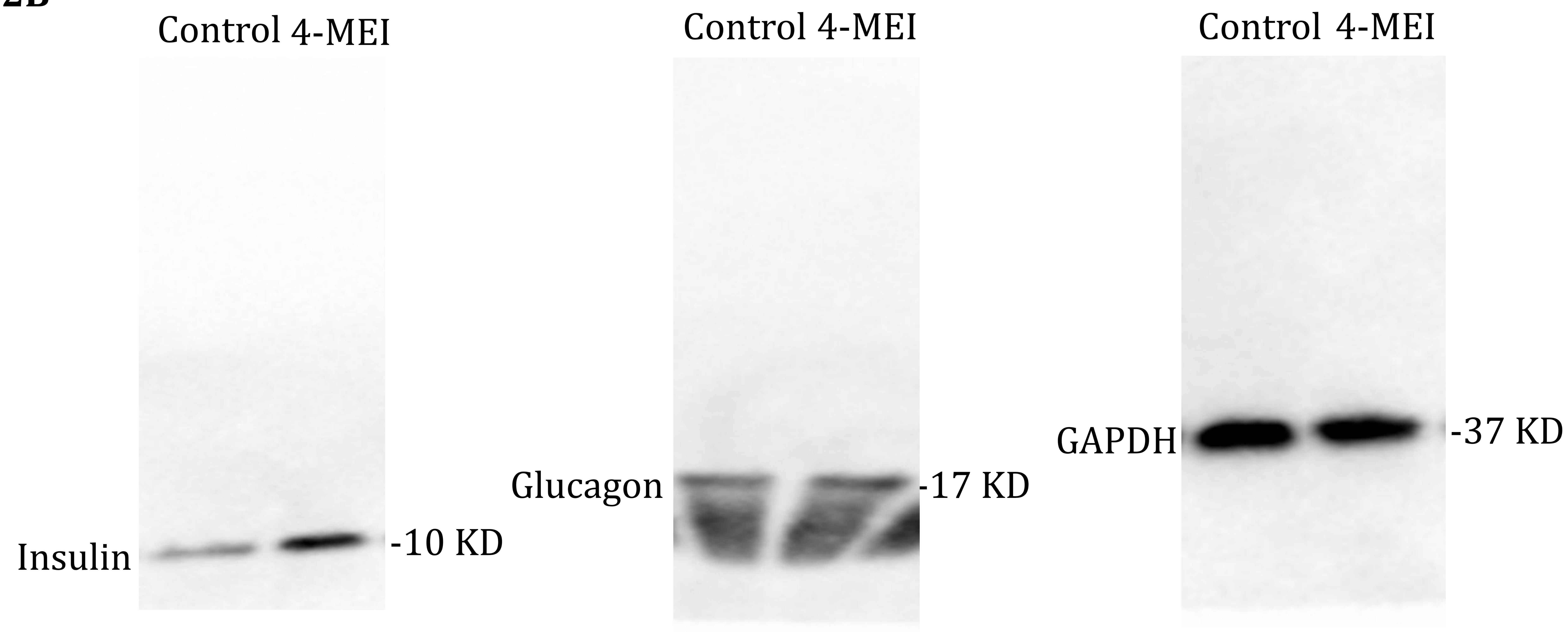

Supplementary Fig. 2: 4-MEI mediated effect in glucose anabolic hormone. S2A. IHC for glucagon in pancreatic tissue sections (400X). S2B. Full length blots of the cropped blot images represented in figure 2C. Experiments were repeated twice.

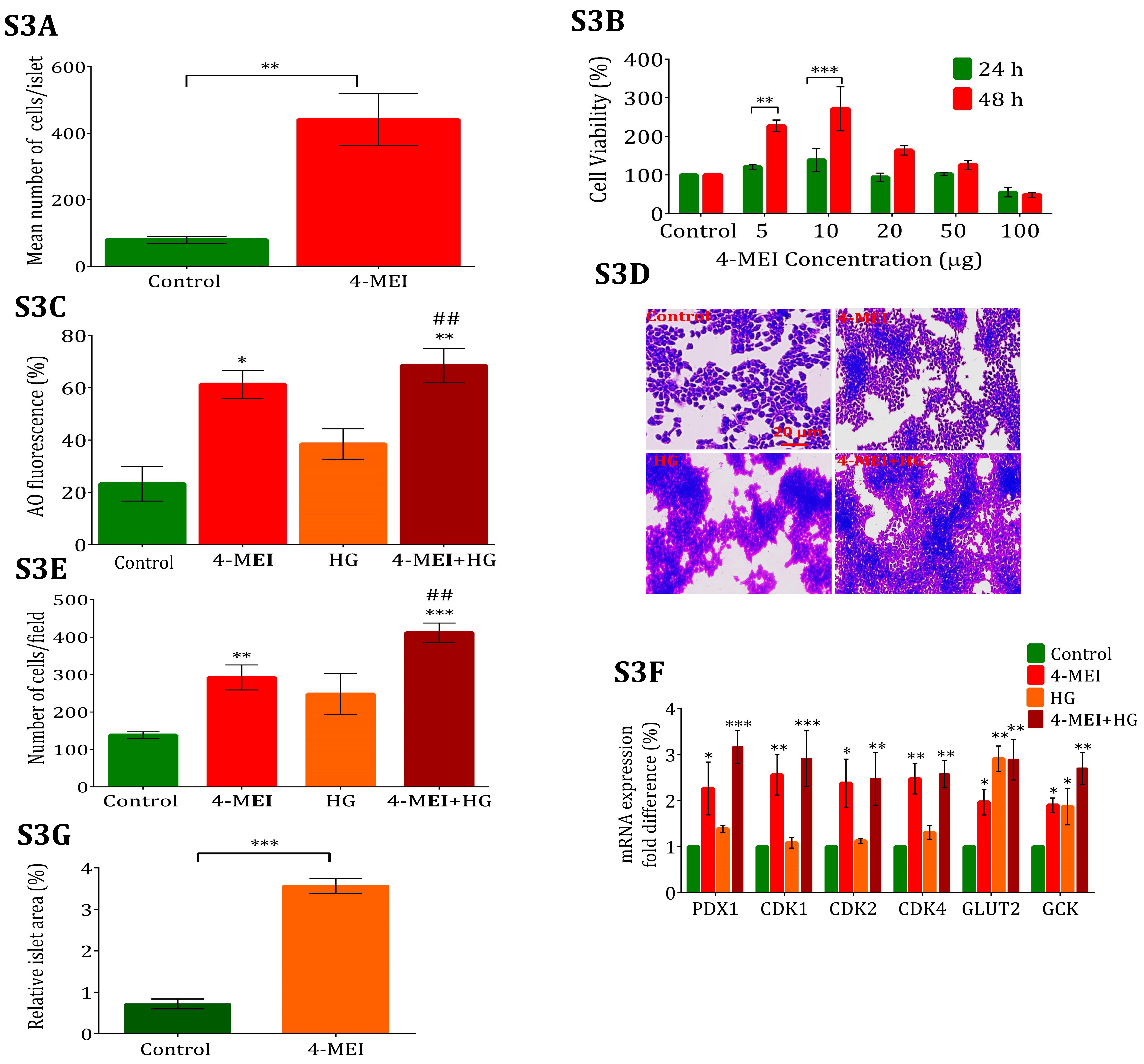

Supplementary Fig. 3: 4-MEI induced pancreatic beta cell hyperplasia. S3A. Cell count showing endocrine cell hyperplasia of mice pancreatic histopathological sections, S3B. *In vitro* cell proliferation and viability determination by MTT. S3C. AO quantification, S3D. *In vitro* hyperplasia determination by CV staining, S3E. Quantitation of CV stained cells, S3F. *In vitro* expression changes of islet cell specific and cell cycle regulatory genes by *q*PCR. S3G. Relative islet area in the pancreatic tissue sections of control and 4-MEI group animals quantified using insulin antibody. * is used to compare experimental groups with the control and # is used for the comparison between HG and 4-MEI+HG groups. Error bars represent mean ± sem; **** $P< 0.0001$, *** $P<0.001$, ** $P< 0.01$, $P< 0.05$/ #### $P< 0.0001$, ### $P<0.001$, ## $P<$ One and Two-way ANOVA with Bonferroni correction. All *in vivo* experiments were repeated twice and *in vitro* experiments were performed with biological triplicates and technical duplicates.

S3G

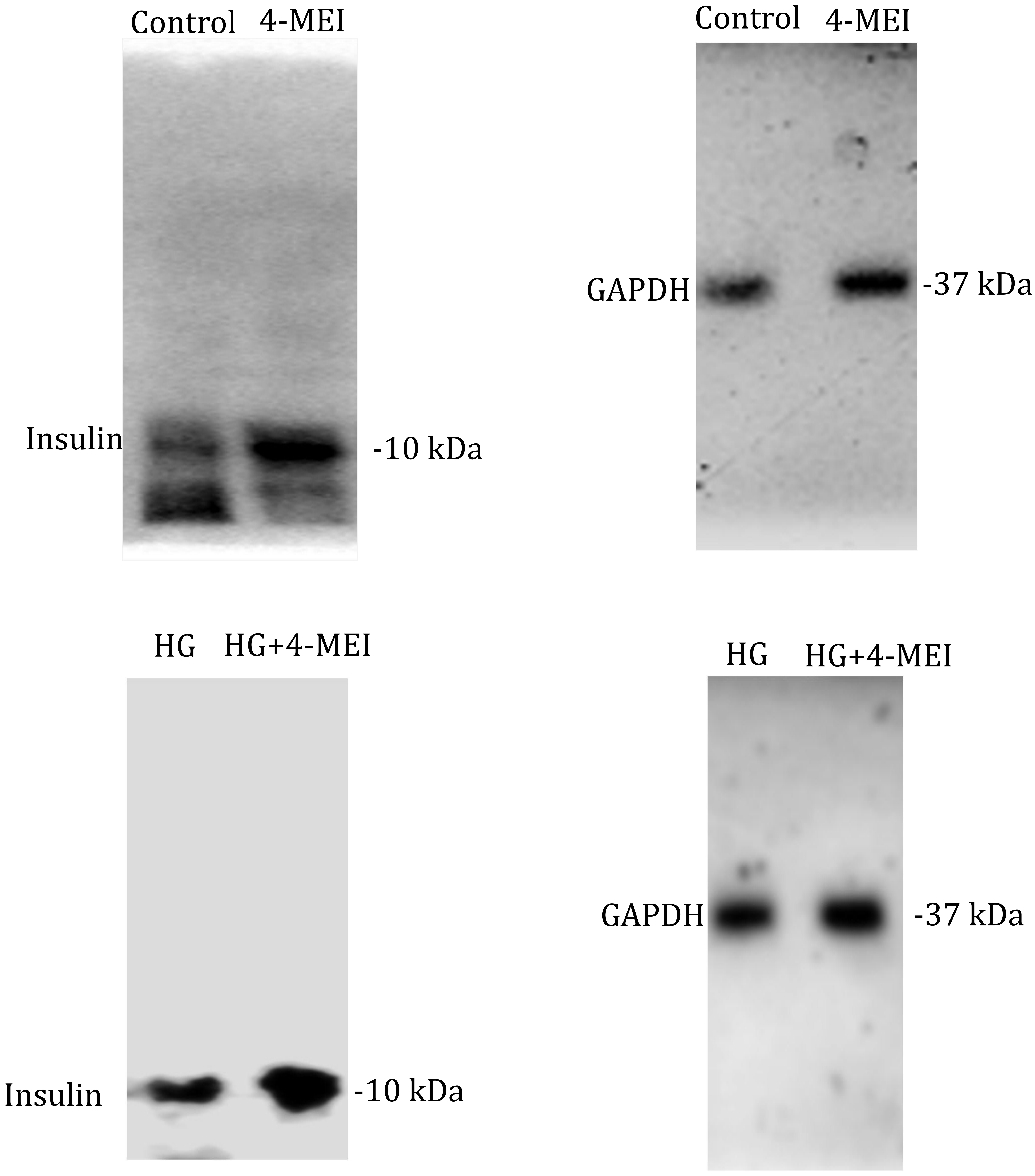

Supplementary Figure S3G: Full length blots of the cropped blot images represented in figure 3D.

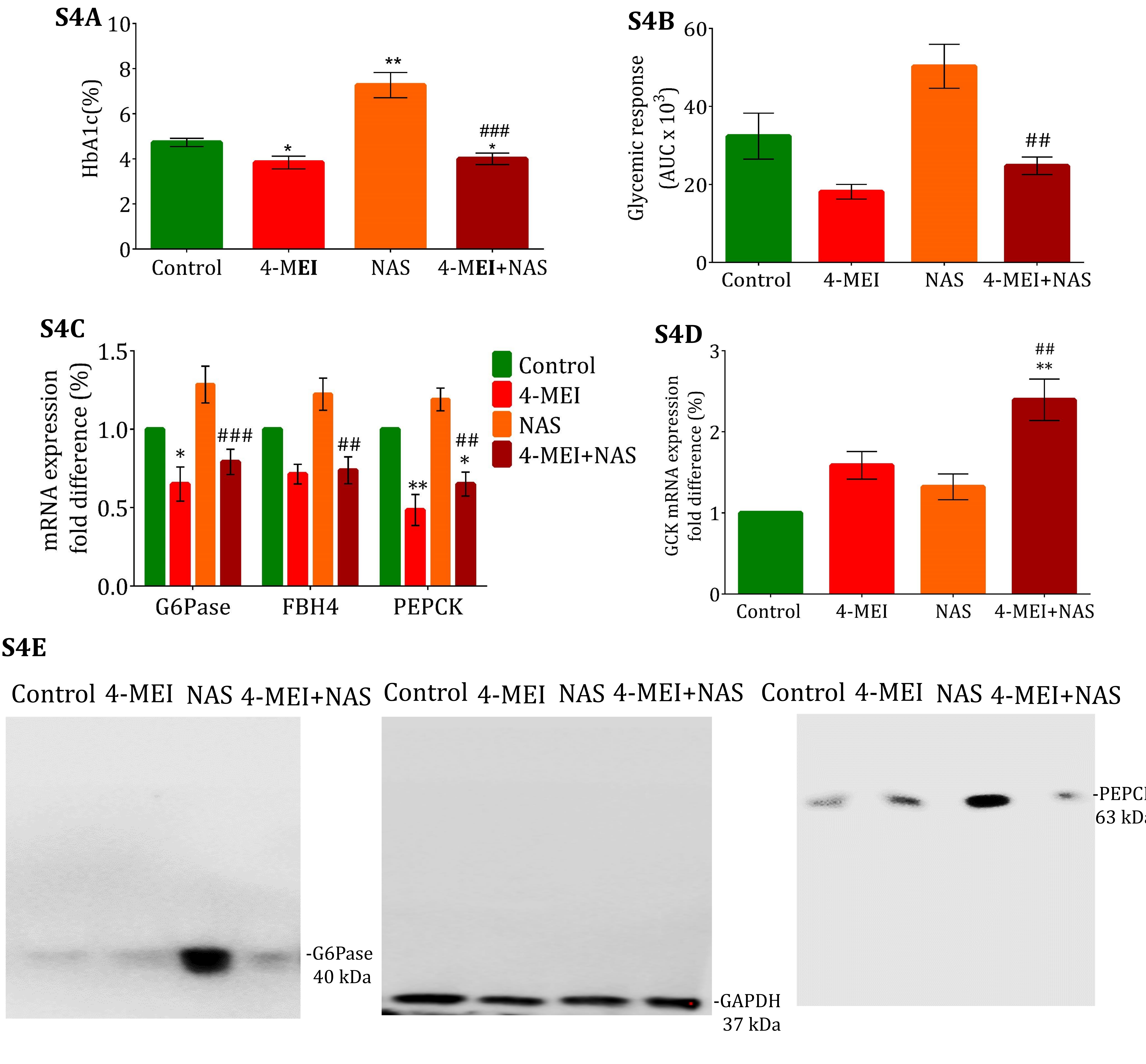

Supplementary Fig. 4: 4-MEI mediated changes in glucose anabolic and catabolic pathways. S4A. HbA1c levels in mice during chronic NAS, 4-MEI and 4-MEI+NAS consumption, S4B. AUC representation of ipPTT, S4C. Gene expression changes of regulatory genes in gluconeogenesis during NAS, 4-MEI and 4-MEI+NAS consumption, S4D. Change in GCK gene expression of NAS, 4-MEI and 4-MEI+NAS treated mice. S4E. Full length blots of the cropped blot images represented in Fig. 4C. * is used to compare experimental groups with the control and # is used for the comparison between NAS and 4-MEI+NAS groups. Error bars represent mean ± sem; **** $P< 0.0001$, *** $P<0.001$, **$P< 0.01$, $P< 0.05$/ #### $P< 0.0001$, ### $P<0.001$, ## $P< 0.01$. Two-way ANOVA with Bonferroni correction and two-sided unpaired Student *t*-test. Experiments were repeated twice.

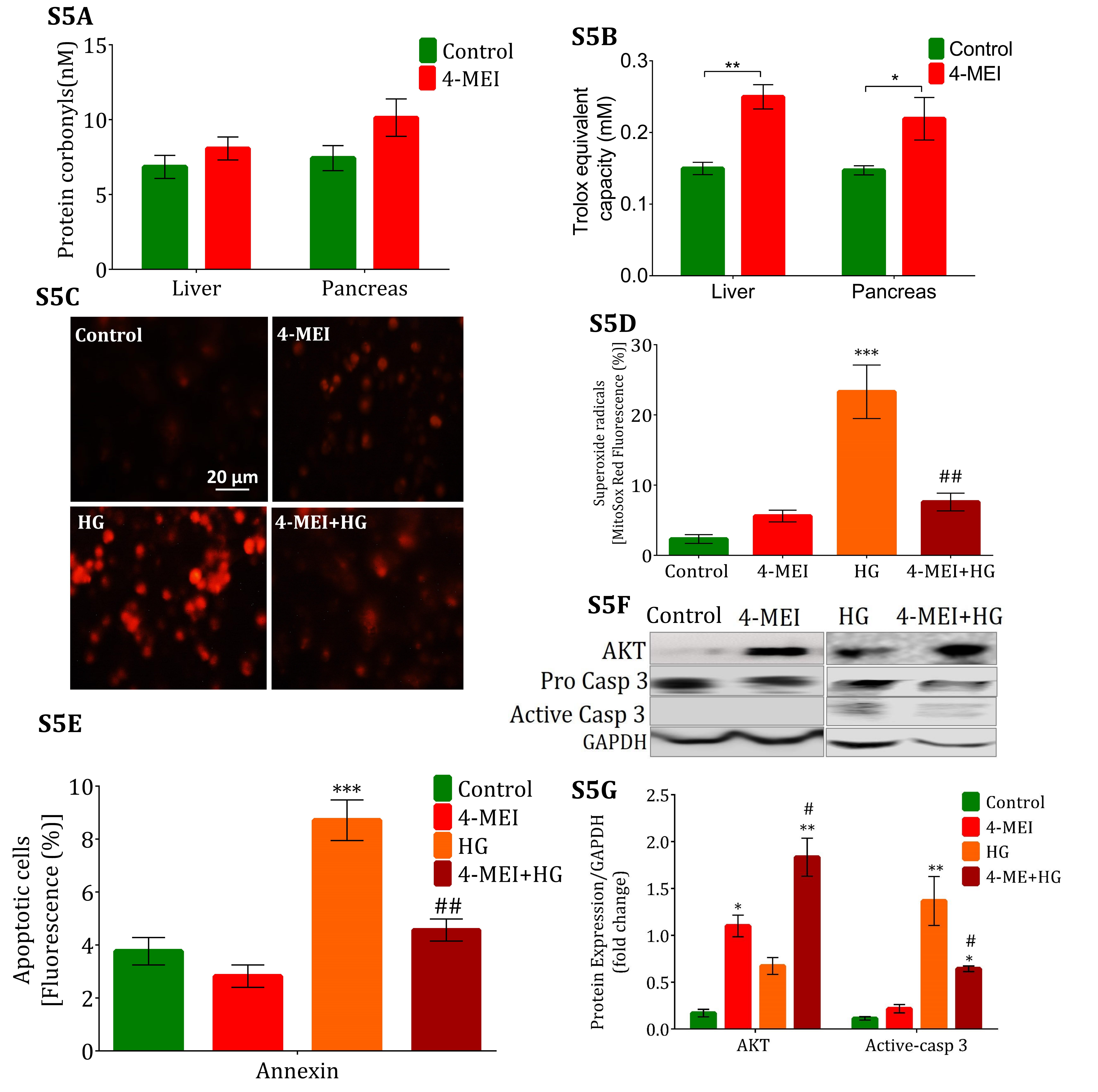

Supplementary Fig. 5: Effect of chronic 4-MEI consumption on ROS generation and cell death. S5A. 4-MEI induced protein carbonylation, S5B. 4-MEI induced change in total antioxidants, S5C. *In vitro* hyperplasia induced mitochondrial O_2^- generation; mitosox red staining, S5D. Quantification of O_2^- , S5E. Quantification of Annexin V stained apoptotic cells , S5F. In *vitro* AKT and Caspase blots, S5G. Quantitation of *in vitro* AKT and Caspase blots. * is used to compare experimental groups with the control and # is used for the comparison between HG and 4-MEI+HG groups. Error bars represent mean ± sem; **** *P*< 0.0001, *** *P*<0.001, ***P*< 0.01, *P*< 0.05/ #### *P*< 0.0001, ### *P*<0.001, ## *P*< 0.01. One-way and Two-way ANOVA with Bonferroni correction. All *in vivo* experiments were repeated twice and *in vitro* experiments were performed with biological triplicates and technical duplicates.

S5H

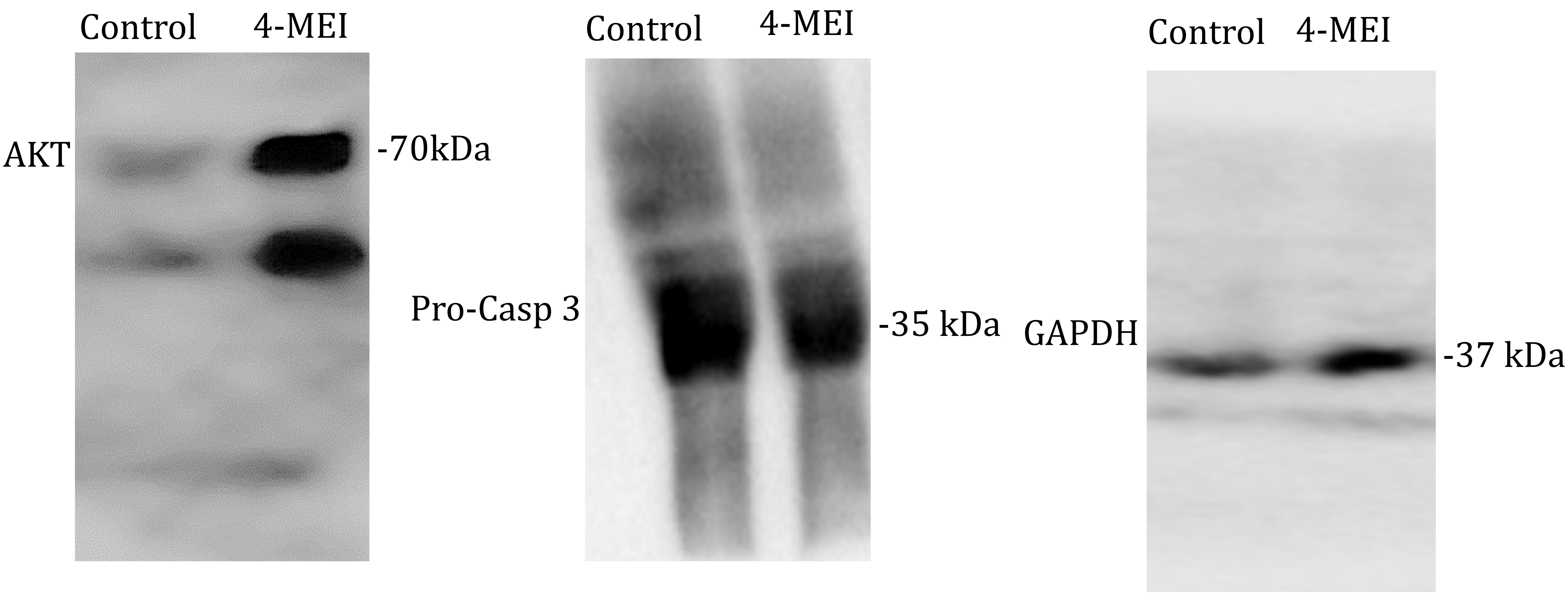

Supplementary Figure S5H: Full length blots of the cropped blot images represented in Figure 5C.

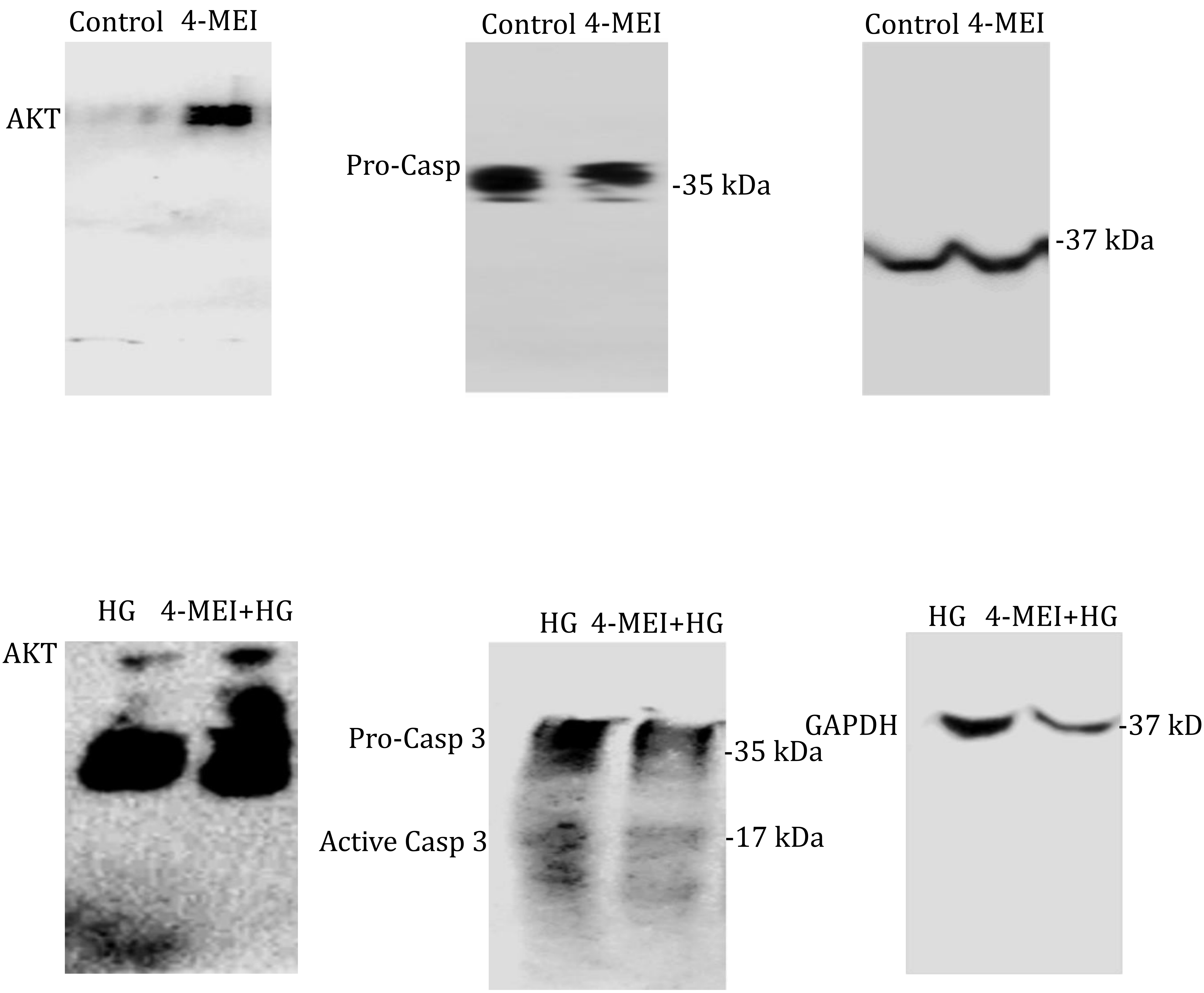

Supplementary Figure S5I: Full length blots of the cropped blot images represented in Supplementary Figure S5F.

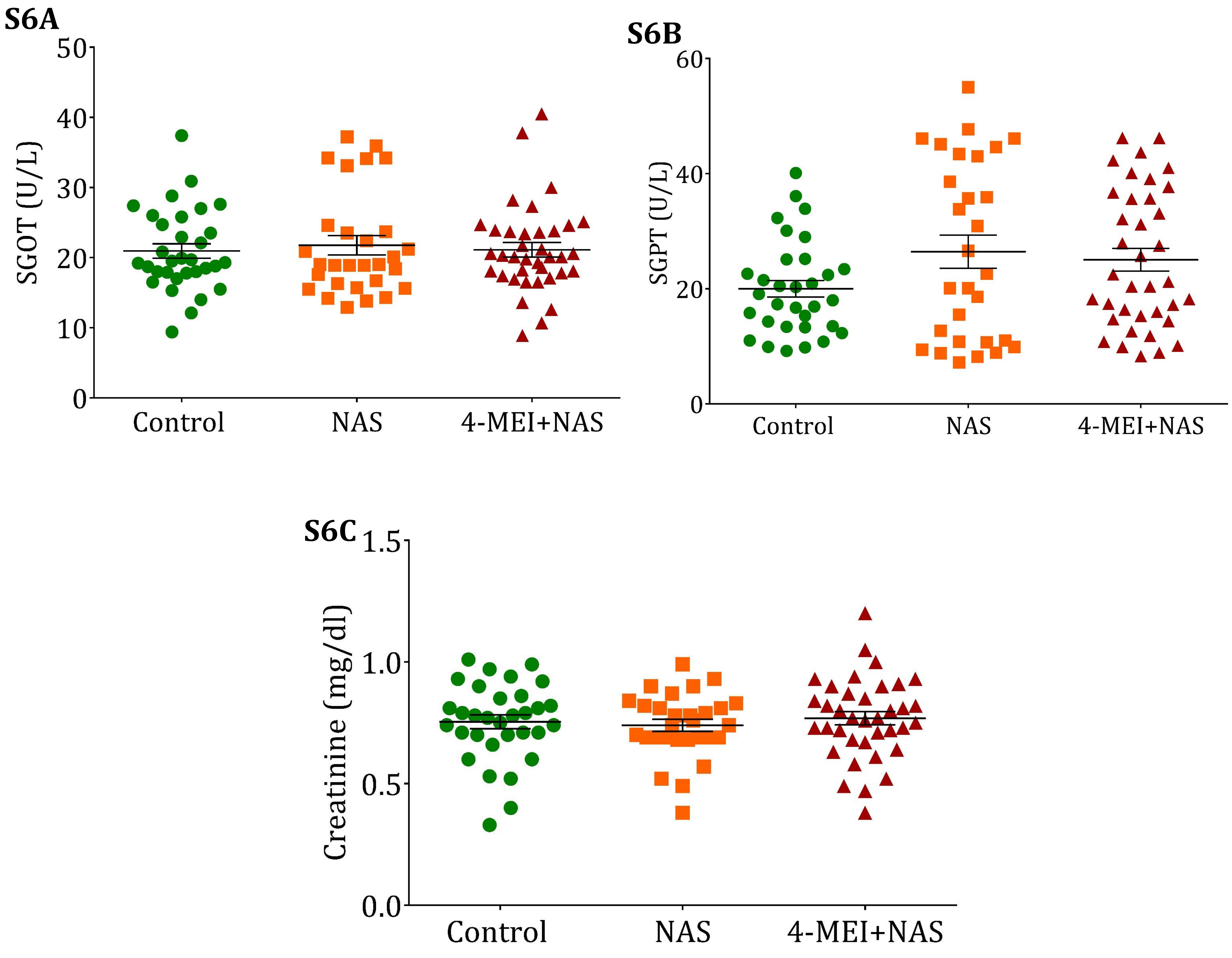

Supplementary Fig. 6: Low dose 4-MEI consumption not induced damage to detoxifying organs. S6A. SGOT for humans, S6B. SGPT for humans, S6C. Creatinine levels of humans. Error bars represent mean ± sem; **** $P< 0.0001$, *** $P<0.001$, **$P< 0.01$, $P< 0.05$. One-way ANOVA with non-parametric Kruskal-Wallis test.

Supplementary Table.1: Primers used for the study

mRNA	Primer Sequence
PDX1-F	ACAAATACATCTCCCGGCCC
PDX1-R	GTCACCGCACAATCTTGCTC
CDK1-F	AAGAAGCTTCCGGTGGTCTG
CDK1-R	AGTGACCGGTTTATCCTGGC
CDK2-F	CAGTTCGGAGGGAAGGACC
CDK2-R	CCCTTACCTCCTTCCTTTGGC
CDK4-F	CGTGGCTGAAATTGGTGTCG
CDK4-R	CCAGCTGCTCCTCCATTAGG
GLUT2-F	AGTCACAGCCCATTTCGAGG
GLUT2-R	GTTACAGGCCACTCCACTCC
GCK-F	AAGAAAGCTGAGGCGTGAGG
GCK-R	ACGGACTCAGCACAAAAGGT
G6Pase-F	CAAGGGAGAACTCAGCAAGTCGT
G6Pase-R	ACTGACAGATGCAAAGGGAACT
Pepck c-F	TAAATCCCAATGGGGGCGTC
Pepck c-R	CCCTAGCCTGTTCTCTGTGC
GAPDH-F	CTGAGAATGGGAAGCAGGTC
GAPDH-R	GAAGGGGCAGAGATGATGAC
